# Federated learning in cancer imaging: a comprehensive review and future outlook

**DOI:** 10.3389/fradi.2026.1976540

**Published:** 2026-10-28

**Authors:** Yiming Shi, Wen Li, Jing Cai

**Affiliations:** 1 The Department of Health Technology and Informatics, The Hong Kong Polytechnic University, Kowloon, Hong Kong SAR, China; 2 The Department of Radiation Oncology and Molecular Radiation Sciences, Johns Hopkins University, Baltimore, MD, United States

**Keywords:** cancer, data privacy, deep learning, federated learning, medical imaging

## Abstract

The rapid development of artificial intelligence has promoted the advancement of data-driven AI medical models, bringing transformative changes to the field of traditional cancer imaging by significantly improving diagnostic accuracy. However, despite these remarkable achievements, a fundamental challenge remains: data privacy. The sensitive nature of medical data and stringent privacy regulations often limit data sharing and collaboration between institutions, hindering the development of robust and generalizable AI models. Federated learning has emerged as a promising solution to address this challenge. By enabling multiple institutions to collaboratively train artificial intelligence models without sharing their local datasets, federated learning reduces the privacy leakage risks associated with conventional centralized AI training while utilizing the collective power of diverse medical data. From 2019 to 2026, extensive research has explored the applications of federated learning in cancer imaging, demonstrating its potential to advance the field while preserving patient confidentiality. In this review, we provide a comprehensive overview of the progress in applying federated learning to cancer imaging, highlighting key studies and their contributions. We also discuss the unique challenges facing federated learning, such as data security, data heterogeneity, and communication overhead. Finally, we explore some potential future research directions. This review aims to provide insights into the evolving role of federated learning in cancer imaging.

## 1 Introduction

Medical imaging techniques, including Magnetic Resonance Imaging (MRI), Computed Tomography (CT), ultrasound, dermoscopic images, histopathological slides, and x-rays, are crucial in the detection and diagnosis of cancer. These modalities offer detailed and precise visual information, enabling physicians to identify and pinpoint tumors effectively. The imaging information is also fundamental in devising a comprehensive treatment strategy (1).

Over recent decades, deep learning has made significant strides, showcasing remarkable effectiveness in various cancer imaging applications, including segmentation, classification, detection, prediction, and image reconstruction (2–5). However, traditional deep-learning approaches face challenges in model generalizability. To train an accurate and generalizable model, it is essential to utilize large-scale and heterogeneous datasets, which always demands collaboration across multiple institutions (6). For model training, the conventional approach is to centralize the multiple institutional data on a specific computational server, but collecting patient data from different external institutions raises security and privacy concerns. Cancer imaging data contains patient privacy-sensitive information, such as patient name, sex, age and anatomy, disease status, etc., making it challenge to share the patient data with external institutions because it increases the data leakage risk (7).

Recently, numerous countries have issued privacy protection regulations to protect patient data, such as the European Union's implementation of the General Data Protection Regulation (GDPR), the China Cyber Security Law (CCSL) and the United States with the California Consuer Privacy Act (CCPA) of 2018 (8–10), leading it impractical to share the patient data among institutions for generalizable model training. In this context, federated learning (FL) (11) has been recognized as an effective solution to prevent sharing patient data across institutions. As a distributed machine learning framework, FL allows geographically diverse hospitals to collaborate on model training while keeping raw patient data local, thereby reducing the privacy risks associated with direct inter-institutional data sharing. Considering the benefits of FL in protecting patient privacy, it has garnered widespread attention in the healthcare field (12). In recent years, several review articles have been published in different perspectives. Rieke et al. (13) explored the challenges of applying FL in digital health and emphasized the need for collaborative efforts to address data-driven healthcare requirements. However, the application of FL in medical imaging, which is instrumental in facilitating disease diagnosis and treatment, was not addressed in their discussion. Nguyen et al. (14) discussed state-of-the-art FL designs for intelligent healthcare, including resource-aware FL, secure and privacy-aware FL, incentive FL, and personalized FL (PFL). They also reviewed the application of FL in four crucial medical domains: federated electronic health record (EHR) management, federated remote health monitoring, federated medical imaging, and federated Coronavirus disease 2019 (COVID-19) detection. However, their coverage of FL in medical imaging was limited, only involving prostate cancer classification, brain tumor segmentation, MRI reconstruction, etc. Chowdhury et al. (15) reviewed the application of FL in cancer and oncology, summarizing FL algorithms for cancer analysis, FL frameworks for cancer research, and privacy-preserving FL algorithms. However, they did not discuss recent emerging FL methods, and their discussion focused on a limited range of cancer types. Kenji Suzuki (16) summarized the development of deep learning in medical imaging. He introduced two major deep learning models, namely massive-training artificial neural network (MTAMN) and convolutional network (CNN), and highlighted their applications in medical imaging. However, there was no exploration of FL applications in medical imaging. Rehman et al. (17) reviewed and evaluated research on FL related to privacy, security, heterogeneity, data variability, aggregation, and missing labels. Nevertheless, their discussion of FL in medical imaging was limited, primarily devoted to brain, skin, and breast. Nazir et al. (18) investigated how FL can be used for segmenting and classifying medical images and discussed the security and performance issues of FL and future research directions. Unfortunately, they did not provide an analysis of FL techniques for tasks such as medical image reconstruction and disease detection. Table 1 summarizes their contributions and limitations of the above-mentioned studies.

**Table 1:** Contributions and limitations of recent FL-based review articles in medical domain.

Ref	Published year	Contribution	Limitation
(16)	2017	Explored the applications of deep learning in various medical imaging modalities, with a particular focus on two popular deep learning models: CNN and MTAMN	No discussion of FL application in medical imaging
(13)	2020	Summarized the advantages of FL in digital medicine, such as electronic health records and medical imaging, and discussed the difficulties and considerations that arise when implementing FL in a healthcare setting	Lacked a comprehensive discussion on the FL applications in specific medical domains
(14)	2022	Provided an introduction of advanced FL designs and their applications in healthcare and explored the future directions of federated intelligent healthcare	Limited discussion on the application of FL in medical imaging
(15)	2022	Summarized FL applications in cancer analysis, cancer research, and privacy-preserving FL algorithms	Other emerging methods were not evaluated and only discussed FL in certain aspects of cancer imaging
(17)	2023	Involved a comprehensive analysis of research on FL for medical imaging, specifically on studies tackling privacy concerns, heterogeneity, and data variability	Limited discussion on FL research specifically related to cancer medical imaging
(18)	2023	Discussed FL techniques for medical image segmentation and classification, as well as the security and challenges associated with FL	The focus was solely on the techniques used for medical image segmentation and classification, without providing an analysis of FL techniques for tasks such as medical image reconstruction and diagnosis

Although numerous reviews exist, there remains a notable lack of a detailed survey dedicated specifically to exploring the emerging applications of FL in cancer imaging. Therefore, this review aims to meticulously assess the latest developments in the application of FL in cancer imaging. Distinct from the general scope of existing reviews, our analysis delves deeply into the use of FL across medical imaging for the 11 most common cancer types according to its global incidence rate, drawing on publications from 2019 to 2026. The contributions of this review are as follows:

1) We explore in detail the application of FL in cancer imaging, covering 11 common types of cancer and various medical imaging modalities;
2) We identify the main challenges of FL in cancer imaging and analyze potential solutions to address these challenges;
3) Finally, we offer possible future directions to advance the role of FL in cancer imaging.

### 1.1 Structure of the survey

This article is divided into five sections. In Section 1, we present the introduction and objectives of this review, Section 2, we provide a detailed explanation of the definition and process of FL. Section 3 reviews the applications of FL in medical imaging for the 11 most common cancer types. In Section 4, we discuss the challenges faced by FL in the field of cancer imaging and provide our perspectives in future directions. Finally, in Section 5, we summarize the aforementioned content.

## 2 Federated learning

### 2.1 Definition of federated learning

FL is a novel decentralized distributed machine learning paradigm, which was first proposed by Google in 2017 (11). FL addresses the limitations of traditional training paradigms, such as centralized training, in privacy-sensitive domains like healthcare. It fundamentally differs from both local training and centralized training. In local training, models are trained exclusively on isolated local data without inter-institutional communication, but often suffering from insufficient data volume to achieve high accuracy and generalizability. In centralized training, data from all institutions is collected and stored on a central server. The server aggregates this data to train a single, unified machine learning model, which leverages large-scale data but introduces privacy leakage risks. In contrast, FL enables collaborative learning across institutions without requiring the exchange of patient data by sharing only the locally trained model parameters, thereby reducing the privacy risks associated with direct inter-institutional data sharing. Figure 1 illustrates the workflows of local training, centralized training, and FL, while Table 2 summarizes their key distinctions.

**Figure 1:**
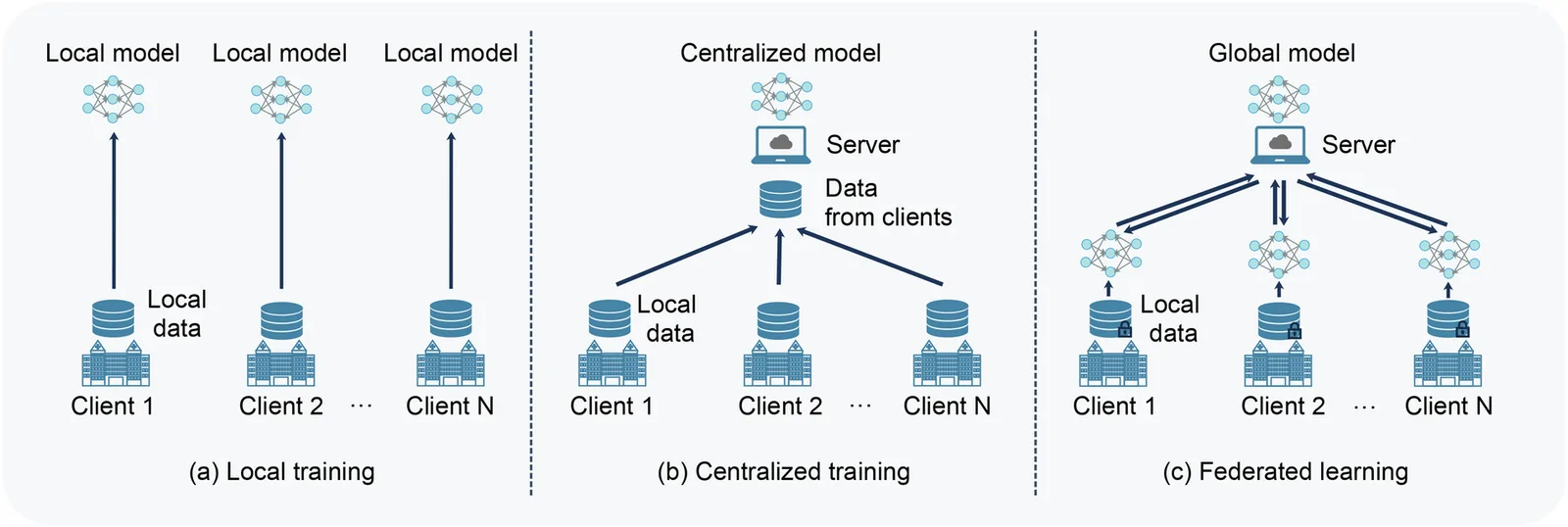
Illustrate the workflows of **(a)** local training, **(b)** centralized training, and **(c)** FL.

**Table 2:** Comparison of training paradigms.

Paradigm	Data Location	Model Update	Privacy Risk
Local Training	Local devices	Independent update	Low
Centralized Training	Central server	Global unified update	High
FL	Local devices	Parameter aggregation, collaborative update	Low

The basic architecture of FL involves two roles: a server and clients. Clients are participating entities, each maintaining a private local dataset that is never shared externally. The server, acting as a coordinator without local data, facilitates the FL process. The workflow comprises two iterative phases: model training and model aggregation. Clients receive a global model distributed by the server and fine-tune it utilizing their own data in the training phase. In the aggregation phase, clients upload their updated local model to the server. The server then aggregates these parameters to refine the global model, which is subsequently redistributed to all clients. This cyclic process constitutes one FL iteration. The training process concludes when the global model achieves convergence following a specified iterations.

### 2.2 Classification of federated learning in medical applications

In the medical domain, the categorization of FL includes three main paradigms that depend on how data is distributed across sample space and feature space: Horizontal FL (HFL), Vertical FL (VFL), and Federated Transfer Learning (FTL).

#### 2.2.1 Horizontal FL

HFL addresses sample heterogeneity by leveraging diverse client-specific datasets to enhance model generalizability. It is applicable when clients share overlapping feature spaces but possess distinct sample distributions. For instance, multiple hospitals may hold MRI scans of different patients, where the imaging modality remains consistent, but the patient cohorts differ. By aggregating decentralized datasets, HFL mitigates biases caused by localized data scarcity. Typical medical applications include multi-center brain tumor segmentation and classification tasks (19, 20).

#### 2.2.2 Vertical FL

VFL exploits feature complementarity through cross-modal joint modeling to improve model performance. This paradigm applies when clients share overlapping sample spaces but possess different feature spaces. A representative scenario involves integrating a patient's MRI scans from a hospital with their genomic data from a biobank, where the same patient cohort is analyzed across heterogeneous data modalities. Medical applications include radiogenomic studies (21) and multimodal diagnostic models combining imaging and clinical data (22).

#### 2.2.3 Federated transfer learning

FTL enables cross-domain knowledge transfer via domain adaptation techniques, facilitating model reuse when clients exhibit non-overlapping sample and feature spaces. For example, Hospital A may hold MRI scans of Patient Group A, while Hospital B possesses CT scans of Patient Group B, with neither same patients nor imaging modalities. FTL bridges such domain gaps, allowing knowledge transfer between modalities (e.g., CT-to-MRI synthesis) and patient populations. Medical applications include cross-modality image reconstruction from data-rich domains (23).

## 3 Review

This section reviews the latest developments in FL for medical imaging, focusing on 11 major cancer types. The advancements discussed cover a range of tasks such as classification, segmentation, detection, reconstruction, and generation (see Figure 2).

**Figure 2:**
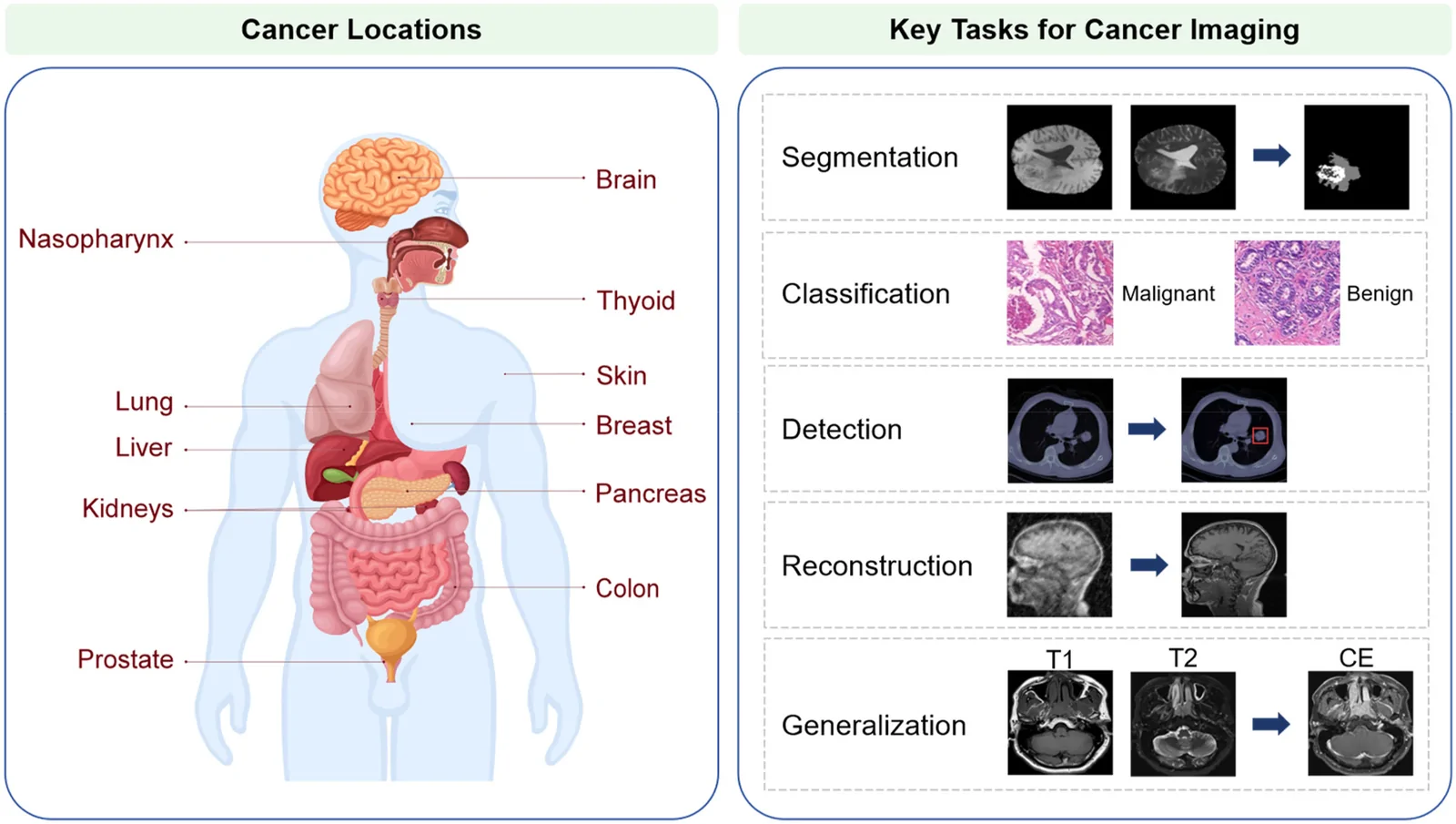
Overview of FL applications in 11 types of cancer research. T1, T1-weighted MRI; T2, T2-weighted MRI; CE, contrast-enhanced MRI. (Left) adapted from “Human anatomy. Infographic elements. Male body” by AlonzoDesign, licensed under iStock Standard License.

### 3.1 Search design

This review examines recent applications of FL in cancer imaging, encompassing both radiological and pathological imaging. Considering the incidence and mortality of different cancer types (24), the review covers lung, breast, skin, prostate, colorectal, kidney, thyroid, liver, pancreatic, brain, and nasopharyngeal cancers.

We searched Google Scholar, PubMed, Web of Science, and Scopus for studies published between January 2019 and August 2026. The search strategy combined terms related to federated learning, cancer, and medical imaging, including terms specific to digital and computational pathology. The following search string was used: (“federated learning” OR “federated deep learning”) AND (cancer OR oncology OR tumor) AND (“medical imaging” OR radiology OR MRI OR CT OR mammography OR dermoscopy OR pathology OR histopathology OR “digital pathology” OR “computational pathology” OR “whole-slide image”).

Studies were considered eligible if they met the following inclusion criteria: (1) peer-reviewed journal articles or high-quality conference papers; (2) the study focused on cancer-related applications involving medical imaging; (3) the study investigated an FL-based method, framework, or privacy-preserving healthcare system; (4) the study was an original research article with a clearly described methodology, experimental evaluation, or system implementation; and (5) the study was published between January 2019 and August 2026. Studies were excluded if they were (1) review articles, editorials, commentaries, or conference abstracts; (2) unrelated to cancer imaging; or (3) did not implement or experimentally evaluate an FL.

The study-selection process followed the PRISMA 2020 guidelines (25), and the complete screening procedure is summarized in the flow diagram shown in Figure 3. The database searches identified a total of 6361 records, of which 68 studies were ultimately included after screening and eligibility assessment. The distribution of the included studies by cancer type is presented in Figure 4.

**Figure 3:**
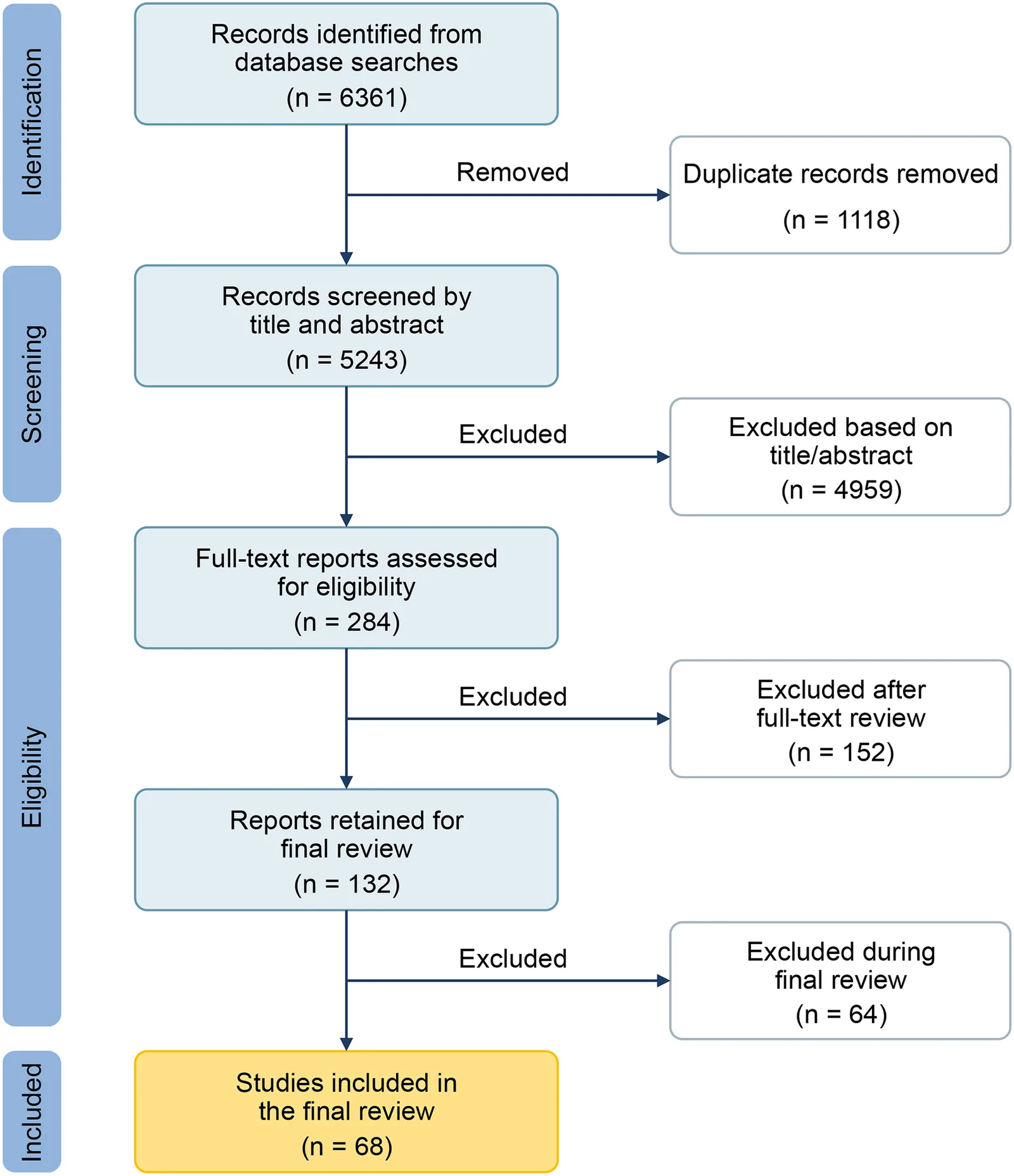
PRISMA 2020 flow diagram of study selection process.

**Figure 4:**
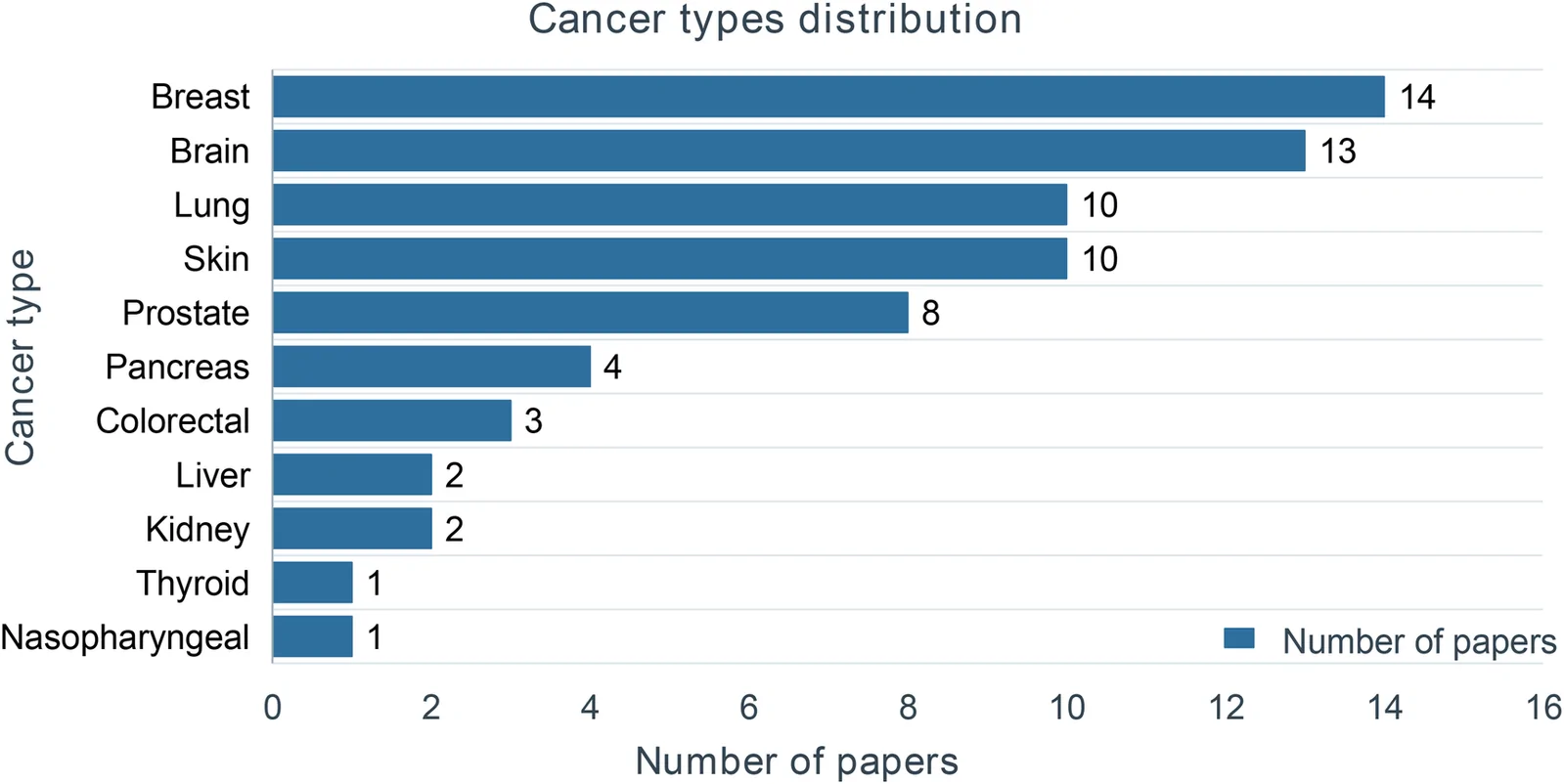
Number of papers on different types of cancer.

### 3.2 FL in brain cancer

Brain tumors comprise a heterogeneous group of neoplasms that arise within the brain and may be benign or malignant. Among malignant primary brain tumors, glioblastoma is the most common in adults and is characterized by aggressive growth, frequent recurrence, and poor prognosis, making it one of the most clinically challenging primary brain tumors (26). The applications of FL in brain cancer include tumor segmentation, tumor classification, and MRI reconstruction, which were summarized in Table 3.

**Table 3:** FL applications for brain cancer. Detailed characteristics of the included studies are provided in Supplementary Table S1.

Task	Ref	Modality	FL method	Dataset	Model	Main result
Segmentation	(28)	MRI	FedAvg	BraTS 2018	U-Net	Dice 0.89, Sens. 0.92, Spec. 0.96
	(20)	MRI	Synchronous weighted FedAvg	BraTS 2018	3D segmentation network	FL achieved performance comparable to centralized training.
	(32)	MRI	FedAvg	BraTS 2017; MDACC and WashU	U-Net	BraTS: Dice 0.86; WashU: Dice 0.77; MDACC: Dice 0.82
	(33)	MRI	SplitAVG; SplitAVG-v2	BraTS 2017	U-Net	Dice: 84.6%
	(34)	MRI	FL	BraTS 2020	3D U-Net	Visible-test Dice 0.67; hidden-test Dice 0.62 / 0.61
	(35)	MRI	CFFT	FeTS 2022 / BraTS 2021	3D U-Net	Dice: 0.89, HD95: 5.8749
	(38)	MRI	Vanilla and decentralized FL tools based on MQTT	BraTS 2018; BraTS 2020; private Athens MRI dataset	U-Net	Dice >0.85
Classification	(43)	MRI	FedAvg	UK Data Service dataset; extended Kaggle dataset	CNN ensemble (DenseNet121, VGG19, InceptionV3)	UK dataset: Acc. 91.05%, AUC 0.91; extended dataset: Acc. 93.22%
	(40)	MRI	Average-weight FL	BT-small-2c; BT-large-3c	CNN	Acc. 82% / 96%, F1 87% / 97%
	(19)	MRI	FedAvg; QFedAvg; Ft-FedAvg; Dp-FedAvg	Brain Tumor MRI Dataset	CNN	10 rounds: Acc. 85.55%; 20 rounds: Acc. 84.60%; 30 rounds: Acc. 85.80%
	(39)	MRI	Collaborative transfer-learning FL	Open-source four-class brain MRI dataset	DenseNet201	IID: Acc. 96.39%; Non-IID: Acc. 94.44%
Reconstruction	(46)	MRI	FedGIMP	IXI; fastMRI; BraTS; Bilkent in-house dataset	Unconditional adversarial model	Outperformed the second-best FL method by 3.7 dB in PSNR and 3.0% in SSIM on average.
	(47)	MRI	FL-MRCM	fastMRI; HPKS; IXI; BraTS	U-Net	T1 SSIM 0.94, PSNR 34.14 dB; T2 SSIM 0.94, PSNR 33.13 dB

#### 3.2.1 Brain tumor segmentation

It is crucial for determining appropriate treatment strategies to segment brain tumors accurately. Traditionally, radiation oncologists must manually perform the tumor segmentation. However, manual segmentation is subject to oncologists’ expertise and experience, and it is also time-consuming (27). The development of automated segmentation algorithms can address these challenges. Ullah et al. (28) concentrated on advancing brain tumor segmentation through a FL framework, which allows multiple medical institutions to collaboratively train a U-Net-based model while keeping their data local. They used the MRI dataset from the multimodal Brain Tumor Segmentation Challenge 2018 (BraTS 2018 dataset) (29–31) to evaluate the effectiveness of this method. The experimental results indicate that as participating medical institutions increase, FL markedly enhances performance metrics. Specifically, when the number of institutions rose from 50 to 100, specificity improved from 0.94 to 0.96, and the Dice Similarity Coefficient (DSC) increased from 0.87 to 0.89. These findings highlight FL's ability to leverage decentralized data from diverse sources to improve model generalization and accuracy while reducing privacy leakage risks. This demonstrates FL's suitability for large-scale collaborative medical applications. These outcomes suggested that FL not only boosts performance but also scales efficiently by integrating additional data sources. Moreover, the study indicated that the FL model surpasses traditional CNN and Recurrent Neural Network (RNN) techniques in both accuracy and efficiency. While FL keeps data local, there is a potential risk of indirect data leakage from shared models. To address this, Li et al. (20) in 2019, integrated differential privacy techniques into the FL framework to bolster privacy defenses. They utilized the BraTS 2018 dataset collected from 13 institutions and applied differential privacy techniques to evaluate the impact of privacy technology on model performance. Their results showed that although the differential privacy module does impact the accuracy of the model, the system still performed adequately well, demonstrating the feasibility of the differential-privacy techniques in medical imaging context. Institutional incremental learning (IIL) and cyclical institutional incremental learning (CIIL) are two collaborative learning methods designed to reduce the need for direct data sharing across institutions. Unlike FL, these methods employ distinct training stages. Sheller et al. (32) evaluated all three methods—IIL, CIIL and FL—in the context of a brain tumor segmentation task using the BraTS 2017 dataset (29–31), providing a comparative analysis of their performance and effectiveness. Their results demonstrated that FL could achieve nearly the same model performance as traditional centralized training, with 99% performance of those trained with centrally shared data. They also demonstrated that the FL is more stable than other data-private methods on the heterogeneity data across different institutions.

Data heterogeneity is a common problem in FL. There are many studies devoted to solving this problem. Zhang et al. (33) proposed a new heterogeneous perceptual FL method, Split Averaging (SplitAVG). SplitAVG split the network into two subnets, located at the network predefined layer and server respectively and connected the feature map. This allowed the joint model to learn unbiased estimates of data across institutions. On the heterogeneous BraTS 2017 dataset, the average DSC for the model hosted on the dataset was 85.67%. Among the models, those trained with SplitAVG achieved the highest average DSC result at 84.6%, followed by Federated averaging (FedAvg) with globally shared data at 83.16%, and Cyclical weight transfer at 79.72%. The result indicates that SplitAVG outperformed other FL methods, although with a slight margin compared to the centralized baseline. Tuladhar et al. (34) developed a FL algorithm on the BraTS 2020 dataset (29–31), introducing an innovative approach that incorporated variable local training epochs and a decaying learning rate across federated training rounds, along with an ensemble weight aggregation function. The FL model attained a DSC of 0.674 on visible test data, demonstrating comparable performance to a centrally trained model's DSC of 0.685. Manthe et al. (35) proposed a novel PFL algorithm, termed Clustered Federated Fine-tuning (CFFT), which is designed specifically for brain tumor segmentation. The algorithm resolved the feature shift challenge resulting from the use of diverse scanners and acquisition protocols across various institutions. The method consisted of three parts. First, each participating institution computed radiomic features from multimodal MRI and combined them into vectors. Second, these vectors were clustered on a central server to obtain clusters with similar radiomic profiles. Finally, the global model was trained using federated average (FedAvg) and fine-tuned for each cluster. The proposed method was validated on FeTS2022 dataset (31, 36, 37) and the results showed that CFFT achieved a DSC of 0.8874, demonstrating effective management of feature heterogeneity within the dataset, which is attributable to varying imaging protocols. It also showed potential for enhancing both the performance and personalization of federated models, surpassing traditional FL methods FedAvg that have a DSC of 0.8803.

FL methods are typically simulated on a virtual framework, where multiple virtual clients are run on a single physical machine. This simulation often overlooks the real-world challenges of distributed environments, such as performance degradation due to inconsistent computing power across different medical institutions and the heterogeneity of real-world datasets. To tackle these issues, Tedeschini et al. (38) developed a novel network architecture leveraging the Message Queue Telemetry Transport (MQTT) protocol. This architecture was designed to assess FL performance in an actual geographically distributed setting. It encompassed both parameter server-based FL and average consensus-based fully decentralized learning within the MQTT framework. To counteract the performance loss stemming from the diverse computational capabilities of medical institutions, they proposed four parameter exchange mechanisms: Parameter Server synchronous, Client synchronous (PS-S/C-S), Parameter Server synchronous, Client asynchronous (PS-S/C-A), Parameter Server asynchronous, Client asynchronous (PS-A/C-A), Consensus-Driven (NO PS), Client asynchronous (C/C-A). The researchers conducted a comparative analysis of these mechanisms to determine the balance between FL training efficiency and model quality. The MQTT architecture was validated across five medical institutions in Europe, employing the brain tumor segmentation task as a benchmark. Public datasets BraTS2018 and BraTS2020, along with the private Athen dataset, were used for validation. The findings revealed that compared to the PS-S/C-A, the PS-A/C-A reduced training time by 20% while attaining a DSC of 0.85. Meanwhile, the convergence time for C/C-A was found to be intermediate between the two. Of the four parameter exchange mechanisms, PS-A/C-A emerged as the most effective in balancing performance with training efficiency. The successful implementation of the MQTT architecture across various European countries is pivotal for gauging the scalability and feasibility of FL systems in genuine geographical contexts.

#### 3.2.2 Brain tumor classification

Brain tumors comprise biologically diverse benign and malignant entities with substantially different clinical behavior and prognosis. Accurate tumor classification and early diagnosis are important for guiding appropriate clinical management (39). Techniques such as FL and deep learning have been utilized to enable faster and more precise brain tumor detection. Mahlool et al. (40) conducted an assessment of FL's viability for brain tumor categorization. They selected two public MRI datasets: Brain tumor-small (41), which is a compact dataset aimed at the binary classification of brain tumors, and Brain tumor-large (42), a more extensive dataset designed to categorize brain tumors into four distinct types (Meningiomas, Pituitary tumors, Gliomas, and No Tumor). For the classification process, they utilized CNN and a pre-trained Visual Geometry Group model (VGG-16). FL demonstrated its potential in brain tumor classification, achieving an 82% classification accuracy on the Brain tumor-small and an impressive 96% on the Brain tumor-large. Similarly, Islam et al. (43) delved into the potential of FL for brain tumor classification. Diverging from Mahlool's approach, they conducted a comprehensive evaluation of six CNN architectures: VGG16, VGG19, Inception V3, Dense CNN (DenseNet)121, Residual Network (ResNet) 50, and Xception. From these, the top three performers (DenseNet121, VGG19, and Inception V3) were chosen to develop an average CNN model tailored for brain tumor classification. The team meticulously labeled MRI images with “Yes_Tumor” and “No_Tumor,” drawing from the UK Data Service (44). The study culminated in the discovery that the FL framework's tumor detection accuracy was pegged at 91.05%, slightly trailing behind the 96.68% accuracy of the composite CNN model. Despite this, the researchers maintain a positive outlook on the future advancements of FL in this domain. Ay et al. (19) evaluated four FL methods (FedAvg, Quantum FL with FedAvg (QFedAvg), Fault Tolerant FedAvg (Ft-FedAvg), and FedAvg with differential privacy (Dp-FedAvg)) for brain tumor classification. They collected three public brain MRI datasets (Figshare, Sar-taj, and Br35H) (45).The datasets contain images of Meningioma, Glioma, Pituitary tumors, and non-tumorous brains. Their results demonstrated that the Dp-FedAvg algorithm, which incorporates differential privacy, excelled in safeguarding data confidentiality. In contrast, FedAvg and Ft-FedAvg algorithms emerged as frontrunners in classification precision, achieving accuracy rates of 85.55% and 85.80%, respectively. These findings underscore the balance between privacy protection and classification efficacy within FL methods. Aggarwal et al. (39) innovatively merged transfer learning with FL to effectively classify diverse brain tumor datasets. They used CNN and transfer learning model DenseNet201 for training. The empirical outcomes were promising: the FL attained a remarkable verification accuracy of 96.39% for data that is independent and identically distributed (IID). Even more impressively, for non-IID data, the accuracy remained high at 94.44%. These results indicate that high classification performance was retained under the evaluated federated configurations.

#### 3.2.3 MRI reconstruction

The prolonged scanning time of MRI limits its further clinical applications. Reducing the amount of acquired data can, to some extent, accelerate the imaging process. In recent years, it has been demonstrated that FL can generate reconstructed images from undersampled MRIs that are comparable to fully sampled ones. Elmas et al. (46) proposed FL of Generative IMage Priors (FedGIMP) in 2023, a brain tumor MRI reconstruction method based on FL. FedGIMP's reconstruction process consists of two main stages: first, cross-site training to develop a generative MRI prior, and second, adapting this prior after incorporating the imaging operator. They compared FedGIMP with other advanced FL (FL-MRCM, FedGAN, LG-Fed, FedMRI), and two traditional reconstruction models without FL. The result demonstrated that the FedGIMP outperformed these models. In order to solve the domain transfer problem in MRI reconstruction, Guo et al. (47) proposed a FL-based method called FL-MRCM for MRI reconstruction. They selected four public brain tumor MRI datasets for experiments, namely FastMRI, HPKS, IXI, and BraTS. Through experiments on these four datasets, they showed that FL-MRCM is effective for MRI reconstruction.

### 3.3 FL in lung cancer

Lung cancer is one of the most prevalent cancers globally and is one of the primary causes of cancer related mortality. In 2020, there were over 2.2 million new cases of lung cancer, with approximately 1.8 million deaths worldwide (24). The applications of FL in lung cancer imaging includes lung cancer classification and detection. We detail these applications below, with a comprehensive summary in Table 4.

**Table 4:** FL applications for lung cancer. Detailed characteristics of the included studies are provided in Supplementary Table S2.

Task	Ref	Modality	FL method	Dataset	Model	Main result
Classification	(50)	CT	FedAvg	Lung cancer datasets from five clients	CNN	Acc. > 95%
	(51)	Histopathology	FedAvg; DP-FL	TCGA LUAD/LUSC WSI	MEM model	ε=2.90, δ=0.0001: Acc. 82.3%; external hospital: Acc.70.7%
	(53)	Histopathology	CIT2FR-FL-NAS	LC25000	CNN	Highest-complexity: Acc. 99.96%; minimum-complexity: 92.98%
	(55)	Histopathology	Hyper-MIL	TCGA-NSCLC; PANDA	Attention-based MIL; server hyper-network	TCGA-NSCLC: AUC 0.92, error 0.21, bAcc. 0.86, F1 0.88
	(56)	Histopathology	DCIaaS	LC25000	CNN	Acc. 96.52%
	(57)	CT	FedAvg	Chest CT-Scan Images Dataset（Kaggle）	Lung-AttNet	2/3 clients: Acc. 92%; 4 clients: Acc. 82%; 6 clients: Acc. 67%
	(60)	Histopathology	PFL	LC25000	Convolutional autoencoder	Acc. 100.0%, Sens. 1.00, Spec. 1.00; AUC 1.00
	(61)	CT	FL-CNN	Lung CT datasets from six medical institutions	CNN	Per-client: Acc. 89.02%–92.81%; macro-average: 88.40%–92.67%
Detection	(63)	CT	FedLRes	IQ-OTH/NCCD	ResNet50	Acc. 99.40%, Prec. 98.92%, Spec. 99.36%
	(66)	CT	FBCLC-Rad	Cancer Imaging Archive; Kaggle Data Science Bowl 2017; LUNA16; local dataset	CapsNet	Acc. 99.69%, F1 99.1%, Spec. 99.4%

#### 3.3.1 Lung cancer classification

Histologically, lung cancer is categorized into several types, including small-cell lung cancer (SCLC), lung adenocarcinoma (LUAD), squamous cell carcinoma (SCC), and large-cell carcinoma (LCC), among others. Each subtype has unique features and treatment responses, making accurate classification vital for effective management (48). Traditionally, lung cancer subtypes are identified using imaging, histology, and molecular testing, but these methods have limitations due to sample variability and experience of clinicians (49). Deep learning techniques, particularly CNN, have demonstrated exceptional performance in the precise classification of lung cancer. However, the sharing and collection of histopathological images between institutions have been constrained by privacy concerns, thereby limiting the further application of deep learning in lung cancer classification. To address this issue, research teams led by Nagpal (50) and Adnan (51) have explored the feasibility of combining FL with CNN for lung cancer classification. Nagpal et al. (50) utilize CNN for image classification and employs the FedAvg technique to update local models. To validate this method, they collected data from five institutions, encompassing information on five types of lung cancer: LUAD, SCC, SCLC, LCC, and bronchioloalveolar carcinoma. The model's classification accuracy averaged more than 95% when evaluated across all clients. The integration of FL and CNN appears to be a promising approach for classifying lung cancer, according to these findings. Adnan et al. (51) building on the use of FL and CNN, implemented an additional privacy-preserving measure—differential privacy—to further enhance data protection. They conducted experiments using histopathological images of lung cancer (including LUAD and SCC) from The Cancer Genome Atlas (TCGA) dataset (52). Considering the heterogeneity of data across different institutions, they partitioned the dataset according to the originating hospitals within TCGA to simulate real-world data distribution. The experimental results indicated that the differential privacy FL model achieved a lung cancer classification accuracy of 82.3%, which is nearly as high as the 83.9% achieved by traditional centralized training model.

FL constructs models by collecting updated models or parameters locally, a process that involves substantial computation of model parameters. To address this, Liu et al. (53) creatively combined neural architecture search (NAS) with FL, proposing a convolutional IT-2 fuzzy rough FL model based on NAS (CIT2FR-FL-NAS) for lung cancer classification. NAS reduces the complexity involved by automatically searching for the most suitable network. They conducted experiments on the publicly available LC25000 dataset (54), which contains 15,000 histopathological images of lung tissue. Their experimental results showed that the CIT2FR-FL-NAS model achieved an accuracy of 99.96%, closely approaching the 100% accuracy obtained by the centralized learning model.

Lin et al. (55) proposed a FL framework based on a hyper-network, termed hyper-MIL, for the classification of histopathological images to address privacy and model accuracy concerns. In this framework, the central server uses hyper-MIL to generate weights for local client networks based on embedding vectors that represent client-specific information. This allows the hyper-network to consider the unique data characteristics of each institution and adaptively generate unique model parameters for each client. To further enhance privacy, noise is incorporated into the parameters before being sent to the central server. They validated their method using the public lung cancer dataset TCGA-NSCLC. Compared to traditional FL models (with a AUC of 0.912), the hyper-MIL network demonstrated superior performance in classifying histopathological images of the lung (with a AUC of 0.920). Some studies suggest that during the FL training process, saving the model across nodes or servers may still pose privacy risks. To address this, Peyvandi et al. (56) proposed a blockchain-based decentralized computational intelligence as a service (DCIaaS) to mitigate several security and privacy vulnerabilities associated with conventional federated model sharing. In the DCIaaS framework, models are trained under FL by data providers, with model parameters shared only through smart contracts on the blockchain. DCIaaS achieved a classification accuracy of 96.52% for lung cancer histopathological images, surpassing the standard gradient descent training model (accuracy of 95%).

Saha et al. (57) proposed a lightweight attention-based CNN architecture, Lung-AttNet, and deployed it within a FedAvg-based FL framework for lung cancer detection. Their architecture integrates a Lightweight Global Attention Module, which comprises spatial attention, self-attention, and channel attention blocks. This design enables effective differentiation among lung cancer subtypes from CT scan images. In the FL environment with 10 communication rounds, Lung-AttNet achieved a global accuracy of 92% with both 2 and 3 clients, demonstrating robust model performance while maintaining data confidentiality. Furthermore, the authors employed explainable AI techniques (Grad-CAM (58) and LIM E^59^) to provide visual interpretations of the model's predictions, enhancing the model's trustworthiness and clinical applicability. Their results demonstrate that federated learning-based lung cancer classification can achieve performance comparable to or even superior to centralized learning (91.5% accuracy).

Medical images collected from different institutions are typically non-IID, potentially reducing the performance of the global model trained using FL. To address this, Ayekai et al. (60) proposed a PFL method for lung cancer prediction. The PFL approach addressed the challenge of non-IID data by customizing models for each client. Their PFL method comprises two main components: client-specific autoencoders and client model clustering. Each client independently trains an autoencoder on its local data to capture the unique data distribution effectively. The server then clusters clients based on the weights generated by each client's autoencoder. During model training, the server trains personalized models for each distinct cluster. The framework was evaluated on the LC25000 dataset using an artificially constructed class-imbalanced setting to introduce label-distribution skew. Under this challenging setting, the proposed PFL method achieved an accuracy of 100.0%, with sensitivity, specificity, and AUC all reaching 1.00; in contrast, FedAvg, FedBN, and FedProx each achieved an accuracy of only 33.3%.

Jindal et al. (61) proposed the FL_CNN model to assess lung cancer severity using CT images. In their study, lung cancer severity was categorized into four levels—very low, low, medium, and high—based on percentage ranges defined by the authors, with different severity levels corresponding to different treatment requirements. They trained the FL_CNN model on CT images collected from six medical institutions. Although the FL_CNN model's diagnostic accuracy differed among various medical institutions due to the heterogeneity of local data, its average accuracy remained around 92%. This reflects the potential of combining FL with CNN in diagnosing the severity of lung cancer.

#### 3.3.2 Lung cancer detection

The accurate identification and diagnosis of lung nodules in CT scans is challenging because of their wide range of sizes, locations, densities, and the diversity of their surrounding environments. These complexities often lead to misdiagnoses in clinical practice. Consequently, related studies have proposed algorithms and systems for automatically detecting lung nodules to enhance early lung cancer identification (62). Usharani et al. (63) introduced FedLRes, a FL framework integrated with a ResNet (64) for automated lung cancer detection. The ResNet architecture incorporates residual blocks to mitigate gradient vanishing issues during training, making it widely adopted for deep learning applications. They extended the standard ResNet50 network with an extra fully connected layer to make it suitable for classifying lung cancer. To reduce the need for direct sharing of patient data, the model was trained under a FL setup. A dataset of lung CT images from 1097 patients was collected from two specialized hospitals in Iraq, comprising 120 benign, 561 malignant, and 416 normal cases. This dataset, named IQ-OTH/NCCD (65), was utilized to validate their model. Their method achieved a classification accuracy of 99.40%, significantly outperforming comparative methods (e.g., K-Nearest Neighbours: 85.25%, Deep Feature Discrimination Network: 94.52%) in terms of diagnostic performance. Heidari et al. (66) combined blockchain technology (67) and FL to propose a new framework FBCLC-Rad for malignant lung nodules detection based on lung CT. The Capsule Networks (68) are utilized in their technique to classify different types of lung cancer locally. They employed blockchain technology to ensure the organization's anonymity while employing FL to train global models. The technique was trained and tested using diverse datasets, including the Cancer Imaging Archive (CIA), Kaggle Data Science Bowl (KDSB), LUng Nodule Analysis 16 (LUNA), and a local dataset. The results showed that the technique achieved a 99.69% accuracy in lung cancer detection.

### 3.4 FL in breast cancer

Breast cancer accounts for 11.7% of all cancer cases, making it one of the most prevalent forms of cancer and the most common cancer in women (24). A variety of tests can be employed for diagnosing breast cancer, including breast physical examination, mammogram, biopsy, ultrasound, and breast MRI. In the past few years, advancements have enabled in utilization of deep learning and other methods to analyze medical images for breast cancer detection and diagnosis. In this section, we describe the applications of FL in breast cancer, with a summary provided in Table 5.

**Table 5:** FL applications for breast cancer. Detailed characteristics of the included studies are provided in Supplementary Table S3.

Task	Ref	Modality	FL method	Dataset	Model	Main result
Classification	(73)	Histopathology	FedAvg	BreakHis	ResNet152; DenseNet201; MobileNet-v2-100; EfficientNet-B7	Image-level Acc. 91.06%; patient-level Acc. 91.87%; F1 84.97%
	(74)	Histopathology	FedAvg	BHI, BreakHis	ResNet, GaborNet	BHI: Acc. 86.57%, F1 86.49%; BreakHis: Acc. 86.32%, F1 87.4%
	(75)	Imaging tabular cytological features	FedAvg	Breast Cancer Wisconsin Diagnostic	Fully connected feed-forward neural network	FL + DP: Acc: 96.1%;FL without DP: 97.7%
	(76)	Histopathology	SiloBN	CAMELYON16; CAMELYON17	DCNN	CAMELYON16: AUC 0.94; CAMELYON17: AUC 0.88
	(77)	Histopathology	FedWSIDD	CAMELYON16; CAMELYON17	ResNet50	CAMELYON16: Acc. 90.1%; CAMELYON17: Acc. 81.2%
	(78)	Histopathology	HistoFS	TCGA-RCC; HER2; CAMELYON17	SSL-ViT encoder with an MIL classifier	RCC: Auc 98.11%; HER2: Auc 86.07%
	(79)	Histopathology	FedDFP	CAMELYON16; CAMELYON17; TCGA-IDH	Heterogeneous feature extractors and MIL classifiers	CAMELYON16: Acc. 90.1%;CAMELYON17: Acc. 91.5%; TCGA-IDH: Acc. 94.3%
	(80)	Histopathology	FedHD	CAMELYON16; CAMELYON17; TCGA-IDH	ResNet-50, UNI, PhikonV2, and GPFM feature extractors; CLAM, TransMIL, and ACMIL classifiers	CAMELYON16: Acc. 91.2%;CAMELYON17: Acc. 82.7%; TCGA-IDH: Acc. 84.8%
	(81)	Mammography	HDRO-FL	CBIS-DDSM	DenseNet, E-RNN	Acc. 95.7%; Sens. 95.7%; Spec. 95.73%; F1 95.8%
	(82)	Mammography	FedAvg	Private mammography data from seven international institutions	DenseNet121	Mean within-site Cohen's κ: 0.68
Detection	(93)	Histopathology	FedAvg	CAMELYON16	CNN	4 client: AUC 98.2%; 3 client: AUC 98.03%
Segmentation	(94)	ultrasound	FedMix	BUS; BUSIS; UDIAT	U-Net	Semi-supervised: Dice 80.6%; mixed supervision: Dice 83.2%
	(98)	mpMRI	Cross-domain FL with FedAvg	Breast mpMRI	U-Net	Dice: 0.65
	(100)	Histopathology	TheODen	BCSS; SemiCOL	U-Net	BCSS: Dice 0.76; SemiCOL: Dice 0.75

#### 3.4.1 Breast cancer classification

Breast tumors are categorized as either benign or malignant, depending on their potential impact on human health. Benign tumors remain localized and don't affect other areas of the body, while malignant tumors are cancerous, tend to grow quickly, and can invade surrounding tissues and organs, leading to significant health concerns. Breast cancer specifically refers to malignant tumors originating from breast tissue (69). Clinically, the diagnosis of breast cancer typically requires professional doctors to carefully examine histopathological images to determine the presence of malignant cells. However, this method carries the risk of misdiagnosis due to insufficient experience of the doctors. In recent times, breast cancer classification has benefited from deep learning, as top-performing models now achieve over 90% accuracy (70–72). However, further improvement in accuracy requires more data for model training. Collecting histopathological images of breast cancer across hospitals raises patient privacy concerns. Therefore, some studies (73–82) have employed FL techniques for breast cancer classification to address the conflict between patient privacy and the need for more data.

Li et al. (73) proposed a FL framework comprising three modules: a federated server, federated clients, and a user platform. The federated clients utilize four advanced models—ResNet-152, DenseNet-201, MobileNet-v2-100, and EfficientNet-b7—to train local models. The model parameters are then encrypted using the homomorphic encryption Cheon–Kim–Kim–Song (CKKS) algorithm and sent to the server. The server aggregates parameters using FedAvg and incorporates a task scheduler during federated training to ensure load balancing and improve resource utilization for multi-user access. The user platform provides various prediction services to users. They validated the framework on the task of classifying breast cancer histopathological images, employing the BreakHis dataset composed of microscopic images of benign and malignant breast tumors (83). The FL model achieved a 13.7% higher classification accuracy than models trained independently, demonstrating a significant performance advantage.

To further enhance the generalization of classification models, Agbley et al. (74) proposed a multimodal network based on FL, utilizing GaborNet and ResNet for feature extraction from breast histopathological images. The ResNet network was fine-tuned on a smaller dataset to ensure better feature extraction from limited breast cancer images. They extracted features from each network and employed a custom-built classifier to generate classification results. Their study was conducted on two breast cancer histopathological image datasets (83, 84), achieving accuracies of 86.57% and 86.32% on these datasets, respectively. In comparison, the accuracies of centralized learning were 85.9% and 86.31%. The experimental results demonstrated that the multimodal network based on FL achieved competitive results compared to centralized learning.

Shukla et al. (75) investigated the integration of differential privacy with FL for breast cancer diagnosis using the Breast Cancer Wisconsin Diagnostic dataset. The framework was evaluated with 10 federated clients under both IID and Non-IID data distribution scenarios, employing the FedAvg algorithm for model aggregation. To enhance privacy protection, the authors implemented gradient clipping followed by Gaussian noise injection, with privacy guarantees controlled by the privacy budget parameters (*ε*, *δ*). Their experiments demonstrated that FL combined with differential privacy achieved 96.1% accuracy with a privacy budget of *ε*=1.9, which is comparable to the 96.0% accuracy of traditional non-FL models while providing strong privacy guarantees. The study systematically evaluated the privacy-accuracy trade-off across different privacy budgets, showing that while stronger privacy (lower *ε* values) introduces slight accuracy degradation, optimal privacy budgets can maintain high diagnostic performance. Additionally, the authors compared differential privacy with alternative privacy-preserving techniques including homomorphic encryption and secure multi-party computation, demonstrating that differential privacy offers the most practical balance between privacy strength, computational cost, and scalability for real-world healthcare applications.

Data heterogeneity is a common challenge in FL, and this issue is also present in breast cancer histopathological images. This heterogeneity is often due to differences in staining procedures and scanning equipment across different medical institutions and imaging artifacts. Andreux et al. (76) proposed a novel FL method called SiloBN to address this. This approach incorporates batch normalization (BN) layers and improves the model's ability to handle diverse data by sharing parameters across certain BN layers, which also helps minimize the potential for privacy risks. They tested the SiloBN method on the breast cancer classification datasets Camelyon16 (85) and Camelyon17 (86). The experimental results showed that, compared to traditional FL methods such as FedAvg and FedProx, SiloBN demonstrated higher robustness and classification accuracy on in-domain heterogeneous data and better generalization ability on out-of-domain data. Jin et al. (77) introduced FedWSIDD, a FL framework for breast cancer whole-slide image (WSI) classification. Rather than aggregating model parameters or gradients as in conventional FL, FedWSIDD employs WSI dataset distillation to generate compact synthetic slides from local histopathological data, which are subsequently shared and aggregated through a central server. To reduce inter-institutional variation in staining, stain normalization is incorporated into the distillation process. The framework was evaluated on the CAMELYON16 and CAMELYON17 datasets, achieving average classification accuracies of 90.1% and 81.2%, respectively, and outperforming several conventional and personalized FL methods. Under heterogeneous local model settings, FedWSIDD also showed greater stability than the evaluated personalized FL approaches. These findings suggest that dataset distillation provides a communication-efficient strategy for federated WSI analysis while accommodating heterogeneous local model architectures. Raswa et al. (78) proposed HistoFS, a federated style-transfer framework to address feature heterogeneity in WSI classification. HistoFS introduces pseudo bag styles to capture multiple morphological style distributions within individual WSIs. In addition, the framework incorporates an authenticity module to preserve diagnostically relevant regions of interest during style transfer, thereby reducing the risk of altering important pathological information. Evaluated on the RCC and HER2 datasets, HistoFS achieved average AUCs of 98.11% and 86.07%, respectively, outperforming conventional multiple instance learning methods, local style-transfer methods, and other federated style-transfer approaches. These findings indicate that modeling intra-slide style diversity while preserving diagnostically relevant regions can effectively alleviate feature heterogeneity in federated computational pathology. Cong et al. (79) proposed Federated Deep Feature Prompting (FedDFP) to address model heterogeneity, communication overhead, and privacy concerns in federated WSI classification. FedDFP introduces lightweight, client-specific learnable prompts that operate directly on patch-level WSI feature embeddings. During federated training, only the prompt parameters are transmitted and aggregated, while the feature extractors and multiple instance learning classifiers remain local to each institution, substantially reducing communication costs. FedDFP was evaluated on CAMELYON16, CAMELYON17, and TCGA-IDH for breast cancer metastasis detection and glioma IDH classification. Compared with local training, the method achieved mean AUC improvements of 0.11–0.13 and gains of up to 0.10 over competing FL approaches. It also reduced the number of communicated parameters by more than 99.9% and converged approximately two to four times faster than conventional FL methods. These results demonstrate that parameter-efficient prompting can provide a flexible and scalable approach to federated digital pathology. Jing et al. (80) further investigated federated learning under heterogeneous feature extractors and multiple instance learning architectures and proposed Federated Heterogeneous Distillation (FedHD). FedHD introduces a Gaussian mixture feature distillation framework with a one-to-one distillation strategy for each WSI, enabling the complex morphological distributions and rich feature representations of individual slides to be better preserved. The synthesized data are shared across institutions and progressively incorporated into local training through a curriculum learning strategy to enhance model generalization. FedHD was evaluated on CAMELYON16, CAMELYON17, and TCGA-IDH under heterogeneous local model configurations, achieving average classification accuracies of 91.2%, 82.7%, and 84.8%, respectively, and consistently outperforming several personalized FL and distillation-based methods.

Mammography is suitable for early lesion screening, but dense breast parenchyma can hinder disease diagnosis (87). To address this, Kumbhare et al. (81) proposed a method called HRDO-FL + E-RNN. Their approach involved creating a FL framework that utilizes DenseNet for feature extraction from breast cancer mammogram images. These extracted features were subsequently classified using an Enhanced RNN (E-RNN). The E-RNN model uses the Hybrid Dragon-Rider Optimization (HDRO) algorithm to adjust parameters, such as the number of training epochs and hidden neurons, for improved RNN performance. This optimization helps improve the model's accuracy. The study simulated real FL scenarios using mammographic images obtained from the Digital Database for Screening Mammography (DDSM) (88, 89). Compared to other advanced models, their model's accuracy improved by at least 1.5%, demonstrating its effectiveness in diagnosing breast cancer.

Studies have shown that women with higher breast density face a greater risk of developing breast cancer compared to those with lower breast density (90). The established Breast Imaging Reporting and Data System (BI-RADS), which relies on 2D mammography (91), defines four breast density categories: fatty (under 25% glandular), scattered fibroglandular densities (25%–50% glandular), heterogeneously dense (51%–75% glandular), and extremely dense (over 75% glandular). However, clinical assessment of breast density is subjective and varies among individuals, making the development of computer-aided automatic classification models crucial for objective and accurate evaluation of breast density. Roth et al. (82) employed the DenseNet-121 model to classify breast density and set up a FL environment in partnership with seven medical centers. The experimental results showed that the FL-trained model outperformed models trained on data from a single institution by 6.3%. They demonstrated that training breast density classification models using FL in real-world settings significantly enhanced model performance, confirming the effectiveness and potential of FL in multi-center environments.

#### 3.4.2 Breast cancer detection

Breast cancer detection involves identifying cancerous and non-cancerous tissues from medical images and locating lesion areas, which is distinct from breast cancer classification that only differentiates the types of lesions. In some developing countries, such as those in sub-Saharan Africa, breast cancer detection is extremely challenging because of insufficient medical resources, outdated technology, and a shortage of professional pathologists, leading to very high mortality rates from breast cancer (92). To address this, Gao et al. (93) developed a FL-based collaborative diagnosis system for metastatic breast cancer. This system trains a MobileNetV2 model locally and sents the model parameters to the server, using the FedAvg algorithm to aggregate and generate a global model. They conducted experiments simulating FL scenarios across four different institutions using the public Camelyon16 dataset. According to their experimental results, the FL model achieved an AUC of 0.982, outperforming the local models whose AUC were between 0.831 and 0.940. Compared to local models, the FL model could accurately identify small cancerous regions in low-quality images and delineate boundary areas more precisely. This study confirmed that the FL-based approach is effective in identifying metastatic breast cancer, providing an effective solution for regions with limited medical resources.

#### 3.4.3 Breast cancer segmentation

Training deep learning models for medical image segmentation often requires a large number of pixel-level annotated images. However, in real-world scenarios, obtaining a substantial amount of high-quality pixel-level annotated images poses challenges due to the limited number of professional annotators, variations in annotation quality, and patient privacy agreements. To address these issues, Wicaksana et al. (94) proposed a FL-based label-agnostic hybrid supervision framework called FedMix, which allows clients to train segmentation models using any type of annotated data. For clients without pixel-level annotations, the method employs cross pseudo-supervision to generate initial pseudo-labels, thereby accommodating various types of annotated data. Additionally, they introduced a novel adaptive aggregation method to enhance model generalization. During FL updates, FedMix dynamically adjusts the aggregation weights based on the contribution potential of different clients’ model updates, evaluated through training loss metrics. They evaluated the breast tumor segmentation performance of this method using three public breast ultrasound datasets (BUS (95), BUSIS (96), and UDIAT 97). In semi-supervised experiments, FedMix outperformed both FedST and FedRGD, with average DSC of 80.6%, 78.5%, and 74.9%, respectively. In a hybrid supervision setting with the inclusion of bounding box or image-level annotations, the average DSC of FedMix increased to 83.2%. FedMix enhances segmentation model performance while preserving privacy and reducing reliance on pixel-level annotations.

Parekh et al. (98) investigated the feasibility of FL in cross-domain tasks in medical imaging, focusing on multimodal and multi-organ tasks. They designed two distinct experiments: the first experiment involved multimodal organ localization, utilizing Positron Emission Tomography (PET) and CT scan images of 50 patients for kidney localization; the second experiment involved multi-organ tumor segmentation, using mpMRI scans to segment brain lesions in 50 patients and breast lesions in 50 patients (29, 99). The experimental results indicated that FL achieved an average overlap similarity of 0.79 in multimodal organ localization, accurately identifying kidney regions. In multi-organ tumor segmentation, the average DSC was 0.65. Their study preliminarily demonstrated the potential of FL in collaborative training across modalities and organs.

Although most existing FL studies in medical imaging have been evaluated in simulated environments, Babendererde et al. (100) investigated the practical deployment of FL across geographically distributed medical institutions. They developed TheODen, an open-source FL framework for histopathological image analysis, to bridge the gap between methodological development and the real-world deployment of federated WSI segmentation. The framework was deployed across three German university hospitals for histopathological image segmentation using the BCSS breast cancer dataset and the SemiCOL colorectal cancer dataset. Despite differences in institutional hardware, network conditions, and local data distributions, the federated models achieved global average DSC values of 0.764 on BCSS and 0.754 on SemiCOL. Unlike studies in which federated clients are simulated within a single computing environment, this work demonstrates the feasibility of conducting FL over real hospital infrastructure across geographically separated institutions and provides practical evidence supporting the transition of FL from experimental simulation to real-world clinical deployment.

### 3.5 FL in skin cancer

Skin cancer is considered one of the most lethal forms of cancer (101). It consists of three main types, namely basal cell carcinoma, squamous cell carcinoma, and melanoma. Among these, melanoma is the most perilous variant, with a mortality rate reaching 75% (102). Early identification of skin cancer greatly improves treatment outcomes. Dermatologists commonly conduct manual examinations or employ computer vision tools to diagnose skin tumors, which may result in misdiagnosis and prolonged analysis times (103). Leveraging FL-based deep learning techniques can improve the accuracy of classifying skin disease types and evaluating their severity. We describe the applications of FL in skin cancer in this section, with a summary provided in Table 6.

**Table 6:** FL applications for skin cancer. Detailed characteristics of the included studies are provided in Supplementary Table S4.

Task	Ref	Modality	FL method	Dataset	Model	Main result
Classification	(102)	Dermoscopy	Async-FL-CNN	ISIC 2019	CNN	Acc. 93.4%, F1 94.8%, Sens. 94.1%, Spec. 96.3%
	(103)	Dermoscopy	Adaptive federated machine learning	ISIC 2019	CNN	Acc. 95.6%, Prec. 0.95, recall 0.95
	(104)	Dermoscopy	FedML	HAM10000	CNN	Acc. 90.1%
	(108)	Dermoscopy	FedAvg	SIIM, ISIC	EfficientNet	Acc. 83.01%, F1 84.02%, AUC 0.94
	(109)	Dermoscopy	RePFL	HAM10000	ResNet-50	Acc. 87.5%/86.86% under two extreme non-IID settings
	(110)	Dermoscopy	Async-FL	ISIC 2019	CNN, SVM	Acc. > 92%
	(111)	Dermoscopy	Privacy-preserving FL	HAM10000; ISIC 2019	DNN	HAM10000: Acc. 88.27%; ISIC 2019: Acc 83.07%
	(112)	Dermoscopy	FedPerl	ISIC19, HAM, Derm7pt, PAD-UFES	EfficientNet	F1 0.75, precision 0.76, recall 0.75
	(115)	Dermoscopy	Federated contrastive learning	ISIC 2019; AtlasDerm; Dermnet; DarkDerm	ResNet18	Recall 59.23%, Prec. 50.21%
	(119)	Histopathology	FedAvg	Slides of skin lesions from 6 German university hospitals	ResNet18	Federated test set: AUC 0.86; the sixth-hospital external test set: AUC 0.91

Li et al. (104) investigated the use of FL for skin cancer classification and, for the first time, integrated CNN with the open-source FL platform FedML (105). FedML offers standardized implementations of various FL algorithms and a comprehensive toolkit, making it easier for researchers to develop and assess their methods. They trained their model using the HAM10000 dataset (106), which contains images of seven different skin lesions, and explored the effects of changing parameters like client count and training epochs. The experimental results showed that the highest test accuracy for centralized learning reached 98.16%, while the highest accuracy for FL was approximately 8% lower than that of centralized learning. However, this accuracy level is still acceptable, and the approach significantly enhances privacy protection.

Hashmani et al. (103) proposed an adaptive intelligent system based on FL for skin disease detection. This system consists of local edge devices for capturing dermoscopic images and a global server for aggregating and updating local models. It employs an online training and classifier updating mechanism to dynamically adapt to newly added skin diseases. They selected the ISIC 2019 dataset (107) to evaluate their method. Experimental findings indicated that, after being trained on the initial four disease categories, the model reached a classification accuracy of 95.6%. However, after adding four new disease categories, the classification accuracy dropped to 89%, which may be attributed to inconsistencies in data quality encountered during online training. This study is the first to propose an adaptive FL system for skin disease detection that supports the dynamic addition of new disease types and explores potential future applications of this system.

Agbley et al. (108) designed a multimodal FL framework consisting of one server and five clients, utilizing two types of data: skin lesion images and patients’ clinical information. Features from skin lesion images were extracted using a pre-trained EfficientNet, while features from clinical data were extracted using a custom network consisting of linear layers, BN, and Rectified Linear Unit activation. The extracted features were joined together and processed by a fully connected layer to obtain the classification results. They trained and evaluated their model using dermoscopic skin lesion images and clinical data sourced from the SIIM and ISIC datasets (106, 107). The experiments revealed that the accuracy of FL (83.01%) was close to that of centralized learning (83.74%). In terms of sensitivity, FL outperformed centralized learning with 85.98% compared to 82.71%, indicating that FL is better at identifying melanoma. Their study successfully combined multimodal data (images and clinical) with FL, achieving privacy-preserving melanoma detection.

Browne et al. (109) proposed RePFL, a lag-aware personalized federated learning framework for skin cancer classification under severe non-IID data distributions and inconsistent client participation. The framework uses late-stage feature embedding alignment to reduce representation differences while preserving client-specific characteristics, together with a stochastic trace-based Fisher Information Matrix score to estimate the contribution of individual clients. It also employs a semi-asynchronous, lag-aware aggregation strategy that incorporates delayed client updates while limiting the influence of stale information. RePFL was evaluated on the HAM10000 dermoscopic image dataset under two highly non-IID settings and achieved classification accuracies of 87.50% and 86.86%, respectively, outperforming several conventional FL and personalized FL methods.

Ain et al. (110) highlighted a key bottleneck in FL, namely the high communication overhead and slow convergence speed. To address this, they proposed an asynchronous FL method (Async-Fed-CNN-SVM). The asynchronous approach does not require all local models to complete training before updating, which helps improve convergence efficiency. The local models are trained using SVM and CNN. They trained their model using the ISIC-2019 skin disease image dataset (106, 107), which includes eight disease categories. The experimental results showed that the proposed Async-Fed-CNN-SVM method achieved an accuracy of over 92%, outperforming FedAvg-CNN (88.7%) and FedMA-CNN (90.9%). Additionally, compared to these two methods, Async-Fed-CNN-SVM required over 400 fewer communication rounds to reach the same accuracy, significantly improving communication efficiency. Building on Ain et al.'s research on asynchronous FL (110), Yaqoob et al. (102) proposed a new asynchronous FL method called Async-FL-CNN. They divided the CNN into shallow layers for extracting general features and deep layers for extracting task-specific features. The shallow layers receive more frequent updates than the deep layers. By asynchronously updating the shallow and deep layers, they were able to reduce communication overhead while maintaining model accuracy. They validated their method on the ISIC 2019 dataset (106, 107). The proposed method attained a test set accuracy of 93.4%, surpassing other leading techniques, whose accuracies range from 80.2% to 89.8%. Moreover, the model converged to 93% accuracy in just 300 communication rounds, whereas FedAvg required 500 rounds. Compared to Ain et al.'s research, Yaqoob et al. considered the different information content between the model layers and extended the asynchronous technique to different layers of the model, balancing communication efficiency and model accuracy.

Lin et al. (111) focused on privacy protection challenges in the practical deployment of federated learning, proposing a lightweight, privacy-preserving FL framework for skin cancer diagnosis. Their framework integrates Fully Homomorphic Encryption (FHE) and Stochastic Controlled Averaging (SCA) techniques to address data heterogeneity and privacy concerns. To reduce communication overhead, they employed layer-wise Packed Fully Homomorphic Encryption (PFHE) and introduced an FHE-friendly deep neural network architecture. Addressing the common issue of Non-IID (non-independent and identically distributed) data across medical institutions, they incorporated an encrypted SCA mechanism that stabilizes global convergence and reduces gradient variance through control variates, thereby mitigating client drift. They validated their method on the HAM10000 and ISIC2019 dermoscopic image datasets, demonstrating F1-score improvements of 2.2% and 4.0%, respectively, compared to baseline FL methods. Notably, this framework achieved communication overhead reductions of 94.85% and 93.48%, respectively, while maintaining encrypted inference at approximately 17.8 s per sample with less than 2% accuracy degradation compared to centralized plaintext models. These results demonstrate that combining CKKS-based homomorphic encryption with optimized neural network architectures enables practical deployment of privacy-preserving AI in clinical settings.

In the medical field, vast quantities of data remain unlabeled, while acquiring labeled data is both difficult and expensive. Bdair et al. (112) introduced FedPerl, a semi-supervised FL approach designed to enhance skin lesion classification by utilizing both limited labeled data and abundant unlabeled data. Inspired by peer learning, each client randomly selects similar peers from the community and combines their model predictions to generate pseudo-labels, thereby enhancing the utilization of unlabeled data. To further strengthen privacy protection, peer anonymization is applied to the clients. They validated FedPerl using four public skin lesion datasets (ISIC19, HAM, Derm7pt, PAD-UFES) (106, 107, 113, 114). The study showed that models trained with FedPerl achieved performance improvements of 15.77% and 1.8% compared to standard and latest semi-supervised FL methods, respectively. This is the first study on FL for skin cancer classification in scenarios with scarce labeled data, laying the foundation for future explorations of FL in related fields. Unlike the strategy adopted by Bdair et al., Wu et al. (115) tackled the problem of scarce labeled data by introducing a FL Contrastive (FCL) framework. Contrastive learning is a self-supervised learning method that makes it possible to train a model using unlabeled data. This framework consists of two stages. The first stage is joint self-supervised pre-training based on contrastive learning, where two models are trained locally using unlabeled data. One model is the primary model used for classification, and the other is a momentum contrast model, utilizing the MoCo architecture to generate remote features for sharing. The contrastive loss between local and remote features guides the updates to the primary model. In the second stage, the primary model is fine-tuned using a limited quantity of labeled examples. They conducted experiments using four datasets—ISIC 2019 (Caucasian skin), AtlasDerm (brown skin), Dermnet (brown skin), and DarkDerm (dark skin) (116–118)—to simulate real-world skin color distribution. Their method outperformed the baseline across various proportions of labeled data (10%–80%). Their study provides a novel approach for scenarios with scarce labeled data by combining FL and contrastive learning.

Haggenmüller et al. (119) conducted a multicenter study on melanoma diagnosis. They collected 1,025 whole-slide histopathological images of clinically suspicious skin lesions from 923 patients across six German university hospitals. They used a pre-trained ResNet for melanoma classification and compared FL with conventional centralized learning in this context. The study results indicated that on the internal test set, the accuracy of FL (85.79%) was slightly lower than that of centralized learning (90.24%). However, on the external test set, FL achieved higher accuracy (91.26%) compared to centralized learning (90.45%). Moreover, this is the first study to validate the performance of FL in melanoma diagnosis using prospective multicenter data, providing an important reference for the development of privacy-preserving medical models.

### 3.6 FL in prostate cancer

Prostate cancer was the second most common cancer among men in 2020 and the fifth leading cause of cancer-related death (24). Its diagnosis primarily relies on ultrasound-guided biopsies and MRI. With the widespread application of FL in medical imaging, several studies have focused on extending FL to the diagnosis of prostate cancer. In this section, we describe the applications of FL in prostate cancer diagnosis and provide a summary in Table 7.

**Table 7:** FL applications for prostate cancer. Detailed characteristics of the included studies are provided in Supplementary Table S5.

Task	Ref	Modality	FL method	Dataset	Model	Main result
Segmentation	(124)	MRI	FedAvg	Multi-center private prostate MRI dataset	Two-Stage Cascaded U-Net	Dice 0.84, Jaccard: 0.72
	(120)	MRI	FedAvg	Private MRI from UCLA, SUNY Upstate, and NCI; PROSTATEx	3D AH-Net	private test sets: Dice 0.90; PROSTATEx: Dice 0.89
	(125)	MRI	FedAvg	MSD-Prostate; PROMISE12; NCI-ISBI13; ProstateX	AutoML	Dice 0.89
	(129)	MRI	HarmoFL	NCI-ISBI 2013, I2CVB, PROMISE12	U-Net	Dice 94.28%
	(132)	MRI	Personalized retrogress-resilient FL	NCI-ISBI 2013; PROMISE12	Deeplab-v2, ResNet-18	Dice 78.28%, IoU 71.92%
Classification	(137)	MRI	CusFL	PROSTATEx; LocalPCa (private)	Custom network	2-client setting: private/public Avg. Acc. 87.5% / 80.5%; private/public Avg. AUC 95.6% / 88.7%
	(138)	MRI	VAFL	LocalPCa; PROSTATEx	Custom network	8-client VAFL: overall Acc. 0.98; AUC 0.998
	(139)	MRI	FedAvg	TCIA PROSTATE-MRI	SBEROA-optimized ShPyFHNet	Acc. 96.75%, MAP 96.4%

#### 3.6.1 Prostate cancer segmentation

Accurate segmentation of the prostate is fundamental for radiotherapy planning, as it ensures precise boundary delineation. However, manual segmentation is a labor-intensive and time-consuming process, and variations in physicians’ experience can lead to inconsistencies in lesion delineation. Computer-aided algorithms can greatly improve both the speed and precision of segmentation.

Sarma et al. (120) explored the effectiveness of FL using real-world multicenter clinical data. They collected T2-weighted MRI scans from 100 prostate cancer patients at each of three institutions: the University of California, Los Angeles (UCLA), the State University of New York (SUNY) Upstate Medical University, and the National Cancer Institute (NCI). The local models were trained using a 3D Anisotropic Hybrid Network (3D AH-Net), and then aggregated and updated on a cloud server (AWS). They conducted internal and external validations on private test sets from the three institutions and a public dataset (PROSTATEx dataset) (121–123). The FL model achieved DSC of 0.895 and 0.889 on the internal and external datasets, respectively, outperforming all local models. The experimental findings showed that FL was effective in real multicenter prostate MRI segmentation, significantly improving model generalization while providing privacy protection for patient data.

Based on the research by Sarma et al., Shiri et al. (124) attempted to extend FL to a larger number of medical institutions. They developed an FL model for prostate lesion segmentation. The local model training employed a two-stage cascaded U-Net, consisting of an improved 3D U-Net for initial coarse segmentation and a dual-attention 2D U-Net for refined segmentation. The FedAvg algorithm was used to aggregate the local models. They collected MRI images from 400 prostate cancer patients across eight different institutions for their experiments. The results showed that the DSC of FL reached 0.84, surpassing the 0.77 achieved by centralized learning. FL enabled privacy protection through multi-center collaboration while maintaining excellent segmentation performance, providing a viable solution for the clinical automation of prostate lesion segmentation.

Due to the differences in scanning equipment and parameters, heterogeneity is prevalent in medical images, potentially impacting the effectiveness of FL. Roth et al. (125) proposed combining FL with AutoML to address this issue. AutoML can dynamically select the optimal network architecture through training a supernet, thereby achieving model personalization to tackle data heterogeneity. They constructed a supernet comprising various candidate modules, such as 3D convolutions, residual blocks, and 2D convolutions, for dynamic selection. The supernet was trained using FL, and different clients selected the most suitable network architecture from the supernet based on their local data. They evaluated this algorithm on four public prostate MRI dataset (122, 126–128). In the internal test set, the FL combined with the supernet reached an average DSC of 88.91%. After applying local adaptation, this score improved to 89.06%, slightly below the 90.15% achieved by centralized learning, but higher than the 88.14% of the local model. In the cross-institutional test set, their method achieved a DSC of 87.5%, significantly outperforming the local model's 81.4%. The experimental results demonstrated the effectiveness of combining FL with AutoML for prostate segmentation, significantly enhancing the model's generalization and providing a reliable solution to address data heterogeneity.

Similarly, to tackle the problem of data heterogeneity, Jiang et al. (129) categorized the impact of data heterogeneity on FL into two types: local drift, referring to biases in client updates, and global drift, referring to biases in global model updates. Existing research often considers only one aspect of the problem. Jiang et al. (129) were the first to propose a FL method (HarmoFL) that simultaneously addresses both local and global drift. They introduced amplitude normalization, which decomposes images into amplitude, representing low-level features, and phase, representing high-level semantics. By normalizing the amplitude of images from each client while preserving phase information, they reduced update drift between clients while retaining critical structures. Additionally, they designed a weight perturbation strategy to mitigate global drift by interfering with the loss variations of clients, thereby reducing the magnitude of gradient changes. Their experiments were conducted on three public prostate MRI dataset (126, 130, 131). For prostate MRI segmentation, HarmoFL obtained an average DSC of 94.28%, significantly outperforming other advanced FL methods (91.04%–92.75%) and exhibiting the smallest variance among clients. Their method proved effective in handling both local and global drift, Their method proved effective in handling both local and global drift, substantial gains in segmentation accuracy on heterogeneous data.

Chen et al. (132) introduced a novel PFL framework to solve the challenge of class imbalance within data heterogeneity. They introduced the Progressive Fourier Aggregation (PFA) method, which stabilizes the integration of global knowledge by retaining the personalized features of client models and aggregating only the features with high similarity at lower levels. Instead of directly replacing local models with the global model, the approach allows local models to learn from the global model. The global model serves as a reference for optimizing local models, while a Cross-Prototype Alignment (CPA) loss is used to align client and global models. This loss function adaptively adjusts its weights to counteract the effects of class imbalance.

They divided samples from two prostate MRI datasets, NCI-ISBI 2013 (34) and PROMISE12 (126), into five clients to simulate an FL scenario, training and testing their method. The experimental results showed that their method achieved an average DSC of 78.28%, outperforming FedProto (76.47%) and FML (76.92%), with clearer segmentation boundaries.

#### 3.6.2 Prostate cancer classification

In prostate cancer, not all cases require treatment. The Gleason score is widely used in medicine to grade prostate cancer (133). Tumors with a score below 6 are classified as indolent cancers, which grow slowly and pose minimal threat, typically warranting observation rather than immediate treatment. Tumors with a score above 7 are classified as intermediate to high-risk prostate cancers, which pose a significant health threat and usually require prompt and aggressive treatment (134, 135). Clinically, the diagnosis of prostate cancer relies on physicians, and subjective factors may influence different doctors’ diagnoses of the same patient. Recently, numerous research have explored the use of deep learning combined with FL for prostate cancer classification (136).

Wicaksana et al. (137) proposed a novel PFL framework called CusFL. CusFL combines two specialized mechanisms to help each client train customized models while leveraging collective knowledge for optimization. The first mechanism involves feature alignment using the federated model, while the second mechanism allows client models to extract useful features from a joint feature extractor based on collective knowledge. They applied this method to prostate cancer classification and tested it on the PROSTATE (121–123), and LocalPCa datasets. The experimental results showed that CusFL achieved a classification accuracy of 87.5%, outperforming existing FL algorithms (79.0%–86.5%). This is the first PFL framework for prostate MRI image classification, providing a new approach for collaboration on privacy-sensitive medical data.

Yan et al. (138) proposed a variance-aware FL (VAFL) framework. The framework aligns client images within a shared image space to lessen differences between them. Client models use a CycleGAN architecture to convert local images to the target space, incorporating classification loss and reconstruction loss to prevent the loss of information in cancerous lesion areas. They validated VAFL on the classification task of clinically significant prostate cancer (CS-PCa), using a local dataset of 135 patients and a public dataset, PROSTATEx, comprising 188 patients. Under various client number settings, VAFL approached centralized learning's performance and significantly outperformed traditional FL.

Kumar et al. (139) introduced FL-enabled SBEROA-ShPyFHNet, a novel deep learning framework designed to detect and classify the severity of prostate cancer using MRI. This framework employs a multi-stage pipeline that integrates image processing, feature extraction, and advanced optimization algorithms to improve diagnostic accuracy. Specifically, the authors fused a Shepard Convolutional Neural Network (ShCNN) and a PyramidNet with a harmonic analysis network to create the Shepard Pyramid Forward Harmonic Net (ShPyFHNet), which handles the final cancer detection and severity classification. To train ShPyFHNet and optimize its hyperparameters while minimizing computational complexity, they utilized the Secretary Bird Earth Radius Optimization Algorithm (SBEROA). When evaluated against existing deep learning models—including standard CNNs, Deep Transfer Learning (DTL), and Deep Neural Networks—the proposed model achieved a accuracy of 96.75%. Additionally, it demonstrated the fastest computational performance, requiring only 3.686 s to process the test dataset.

### 3.7 FL in pancreatic cancer

Pancreatic cancer is highly deadly and typically detected in its later stages, which makes treatment challenging (140, 141). Early detection of pancreatic cancer is crucial. Pancreatic imaging is essential for both diagnosing and managing diseases. Manual segmentation of the pancreas by radiologists is the standard method for identifying and quantifying pancreatic cancer (142), but this approach is notably time-consuming. Deep learning and FL can be used to explore highly accurate automatic segmentation techniques for pancreatic cancer, an area that is worth investigating. In this section, we describe the applications of FL in pancreatic cancer, with a summary provided in Table 8.

**Table 8:** FL applications for pancreatic cancer. Detailed characteristics of the included studies are provided in Supplementary Table S6.

Task	Ref	Modality	FL method	Dataset	Model	Main result
Segmentation	(148)	CT	DTP, DWA	TCIA; MSD; Synapse	C2FNAS	DTP: Dice 46.1%; DWA: Dice 61.4%
	(144)	CT	Auto-FedAvg	MSD, TCIA, BTCV	3D U-Net	Dice 74.21%
	(143)	CT	Horizontal FL	Abdominal CT images from two multinational institutions	C2FNAS	Dice: C1 pancreas 82.3%; C2 pancreas 65.4%; C2 tumor 46.4%; pancreas average 73.1%
	(149)	CT	FedRNN	MSD, Pancreas-CT, BTCV	Elman RNN	Dice 78.7%

Wang et al. (143) proposed an innovative FL method for pancreatic segmentation. For the neural network architecture, they implemented a coarse-to-fine network architecture search (C2FNAS) and added a variational autoencoder (VAE) branch in the encoder. This setup enhances the model's generalization ability across different datasets by learning more robust data representations. They collected 906 abdominal CT images from two international institutions to evaluate their method. The FL approach resulted in an average DSC of 73.1% for pancreatic segmentation across the two clients, significantly outperforming the local models of the two clients (60.3% and 65.1%). This study is the first to implement FL for pancreatic segmentation in a real-world cross-national scenario, demonstrating the effectiveness of FL in this domain.

Xia et al. (144) proposed a novel Auto-FedAvg method to address the heterogeneity in pancreatic tumor datasets. Auto-FedAvg flexibly adjusts how much each participant's data influences the model, based on the data characteristics and training status. This method learns weights from the data in a differentiable manner and models the aggregation weights using a Dirichlet distribution to automatically find the optimal aggregation weights. They validated the effectiveness of Auto-FedAvg on three public pancreatic CT datasets: the pancreas segmentation subset of the Medical Segmentation Decathlon (MSD) (145), the Cancer Imaging Archive (TCIA) Pancreas-CT dataset (146), and the MICCAI Multi-Atlas Labeling Beyond the Cranial Vault challenge (BTCV) (147). Auto-FedAvg achieved an average DSC of 74.21%, outperforming FedAVG (73.11%) and FedProx (73.29%). This is the first approach to introduce dynamic weights into FL to address heterogeneity in pancreatic tumor segmentation, providing a new perspective for future research.

Building on the research by Xia et al., Shen et al. (148) investigated two FL-based multi-task optimization methods, Dynamic Task Prioritization (DTP) and Dynamic Weight Averaging (DWA), to address the heterogeneity in pancreatic tumor datasets. DTP dynamically modifies the loss function weights assigned to different clients according to the complexity of their tasks, utilizing performance indicators like DSC to dynamically determine client weights. DWA dynamically adjusts the aggregation weights based on changes in client losses, assigning greater weight to clients with larger loss variations, thereby encouraging the global model to focus on more challenging tasks. They used three public datasets (MSD, TCIA, BTCV) (145–147)to simulate three heterogeneous clients: one with only healthy pancreas images, one with only tumor-containing pancreas images, and one with pancreas images among multiple organs. The experimental results showed that DWA and DTP achieved average DSC of 61.4% and 46.1%, respectively, while traditional FL methods FedAvg and FedProx achieved average DSC of 56.9% and 58.5%, respectively. The DWA method led to a significant improvement in pancreatic segmentation by dynamically adjusting the aggregation weights, whereas the DTP method showed slightly less satisfactory performance.

Inspired by the dynamic weights of Shen et al., Deng et al. (149) developed FedRNN, which adapts aggregation weights dynamically throughout the training process. This method employs an RNN to update the aggregation weights for the current round by integrating the previous round's aggregation weights with the current client losses. It effectively incorporates historical information to adjust the weights. They evaluated the performance of FedRNN using three public pancreatic CT datasets (MSD, the National Institute of Health Clinical Center (NIH), BTCV) (145, 147, 150) to simulate three heterogeneous clients with significantly different data volumes. The experimental results showed that FedRNN outperformed FedAvg and DWA, achieving an average DSC of 78.7%, compared to 74.5% for FedAvg and 77.7% for DWA. Another important finding was that as the training process progressed, the aggregation weights of the FedRNN method tended to stabilize, whereas the weights of the DWA method fluctuated significantly. This indicates that FedRNN offers higher stability and generalization in pancreatic segmentation tasks.

### 3.8 FL in other cancers

FL applications on other common cancers, such as colorectal cancer, kidney cancer, thyroid cancer, liver cancer, and nasopharyngeal cancer, have also been investigated but with limited references. These applications are outlined below, with a summary in Table 9.

**Table 9:** FL applications for other cancers. Detailed characteristics of the included studies are provided in Supplementary Table S7.

Task	Ref	Modality	FL method	Dataset	Model	Main result
Colorectal classification	(151)	Histopathology	FedAvg	LC25000	VGG 16 network	Lung: Acc. 99.99%; colon: Acc. 99.72%
	(152)	Histopathology	FedAvg	NCT-CRC-HE-100K	CNN	Acc. 96.04%
	(154)	Histopathology	FedDropoutAvg	TCGA CRC-DX dataset	ResNet18	Independent sites: F1 0.91, AUC 0.95; local test sites: F1 0.85, AUC 0.97
Liver segmentation	(155)	CT, MRI	FedNorm / FedNorm+	LiTS17, 3D-IRCADb-01, Multi-Atlas, SLIVER07, and CHAOS19.	U-Net	CT: Dice 0.96; MRI: Dice 0.94
	(159)	CT	Fed-MENU	LiTS, KiTS, pancreas, spleen, AMOS, BTCV	MENU-Net	Dice 91.14%
Thyroid classification	(165)	Ultrasound	FL	Thyroid ultrasound images from six institutions	VGG19, ResNet50, ResNext50, SE-ResNet50, SE-ResNext50	Internal: Acc. 73.9%–79.5%; external: Acc. 56%–76%
Kidney classification	(166)	Histopathology	Prop-FFL	Kidney Dataset from TCGA	DenseNet121	Acc. 86.7%
	(167)	Histopathology	Weakly supervised FedAvg with Gaussian parameter noise	Kidney Dataset from TCGA	Multiple instance learning-based framework	BRCA: AUC 0.833–0.862; Rcc: AUC 0.974–0.976
Nasopharyngeal synthesis	(168)	MRI	FL-TrustVCE	MRI dataset from 18 institutions	MMgSN-Net	Internal: MAE 23.45, PSNR 33.23; external MAE 30.86, PSNR 31.87

#### 3.8.1 Colorectal cancer

As the third most common cancer worldwide, colorectal cancer constitutes 10% of global cancer cases and has a mortality rate of 9.4% (24). The transformation of polyps in the colon and rectum into colorectal cancer occurs gradually over a long period. Early detection and intervention in the progression of these polyps can significantly improve patient survival rates. Developing computer-aided diagnosis systems is helpful in the early screening of colorectal cancer.

Agbley et al. (151) combined FL and deep learning for the classification of lung and colorectal cancers. This study utilized the LC25000 dataset, which contains 25,000 histopathological images of lung and colorectal cancer. Their method achieved a classification accuracy of over 99% for both lung and colorectal cancers, closely matching the performance of centralized learning models, thereby validating the effectiveness of FL.

Arthi et al. (152) developed a method combining FL and explainable artificial intelligence (XAI) to achieve high-accuracy, privacy-preserving colorectal cancer detection. They utilized 100,000 histopathological images of colorectal tissue provided by Kather et al. (153). Four different CNN networks—VGG16/19, InceptionV3, ResNet50, and ResNeXt50—were tested, with ResNeXt50 ultimately selected for its superior performance. They then constructed a FL framework and integrated Lime, an XAI technique, to visualize the model's decision-making basis. The experimental results demonstrated that FL combined with ResNeXt50 achieved high-accuracy classification (96.04%), closely approaching the performance of centralized learning (99.53%).

Gunesli et al. (154) introduced the FedDropoutAvg approach, utilizing dropout techniques to improve the generalization performance of models used for colorectal cancer classification. During the training process of FL, they randomly dropped some clients to improve the model's robustness. Their study utilized the TCGA colorectal cancer dataset. Experimental findings indicate that the FedDropoutAvg method achieved a AUC of 0.954, significantly outperforming FedAvg (0.945) and FedProx (0.923).

#### 3.8.2 Liver cancer

Liver cancer is the world's seventh most common cancer, with its incidence increasing annually (24). Physicians commonly rely on imaging techniques such as CT and MRI to differentiate between cancerous and normal liver tissue, which helps inform the next steps in patient care. AI-assisted segmentation can quickly and accurately segment tumors, reducing human error.

Bernecker et al. (155) proposed two FL-based modality normalization techniques, FedNorm and FedNorm+. FedNorm implements modality normalization at the client level, while FedNorm + achieves it at the slice level. They evaluated their methods on six public liver segmentation CT and MRI dataset (156–158). The study results showed that the FedNorm and FedNorm + methods improved the average DSC by 5%–20% compared to local models, demonstrating excellent segmentation performance while preserving privacy.

Due to the varying standards for liver segmentation annotations across different institutions, there may be inconsistencies in labels between institutions. To overcome this challenge, Xu et al. (159) developed a FL-based multi-encoder U-Net method called Fed-MENU. They designed multiple sub-encoders to focus on extracting features of specific organs, avoiding interference from unannotated organs, and introduced an auxiliary generic decoder to enhance the distinction of features from different organs. They evaluated their method on six abdominal CT image datasets (liver, kidney, pancreas, spleen, AMOS, and BTCV) (128, 146, 160–164). The average DSC of Fed-MENU across these six datasets reached 91.14%, slightly below the 92.02% achieved by centralized learning. This is the first study to implement FL in joint multi-organ segmentation and effectively address the issue of label inconsistency.

#### 3.8.3 Thyroid cancer

Thyroid cancer is the eleventh most common cancer globally and ranks ninth in incidence worldwide (24). Clinically, thyroid cancer manifests as thyroid nodules. Early detection of thyroid cancer can be achieved through thyroid ultrasound, which helps distinguish between benign and malignant nodules.

Lee et al. (165) investigated the potential of using FL for thyroid classification tasks. They collected 8,457 thyroid nodule ultrasound images from six institutions, including 5,375 malignant and 3,082 benign images. Additionally, they collected 100 ultrasound images from another institution for external validation. The study results indicated that FL performed comparably to centralized learning on both internal and external test sets, with a performance gap of less than 5%. Their research validated the feasibility of FL for diagnosing thyroid cancer in real-world medical settings, demonstrating stable performance even in training scenarios with uneven data distribution.

#### 3.8.4 Kidney cancer

Kidney cancer is the 12th most common cancer worldwide (24). The presence of renal cell carcinoma can be definitively diagnosed through pathological examination (surgery or biopsy). However, analyzing histopathological images is highly labor-intensive and time-consuming. Developing computer-aided diagnosis systems can help improve diagnostic efficiency and accuracy.

Hosseini et al. (166) proposed a FL algorithm for kidney cancer diagnosis. They highlighted a common issue in FL: significant disparities in data volume between different institutions. Traditional FL algorithms tend to favor institutions with larger datasets, which is unfair to those with smaller datasets. To address this, they introduced a proportional fairness strategy in FL, aggregating local models based on the amount of loss reduction and a fairness term. Their method achieved an average accuracy of 86.7% on a public kidney dataset (157) from TCGA, outperforming FedSGD's 82.1%. Additionally, the accuracy of the institution with the smallest dataset improved from 75.3% to 83.5%.

Lu et al. (167) proposed a federated weakly supervised multi-instance learning method to reduce the reliance on pixel-level annotated data. Weakly supervised learning allows model training using only slide-level or patient-level labels. They employed a pre-trained ResNet50 for the classification task of renal cell carcinoma. Building on this, they integrated a gated attention network, utilizing histopathological images and patient prognosis information to construct a renal cancer survival prediction network. They collected a dataset of renal cell carcinoma from TCGA, comprising 937 histopathological slides and 511 patient cases with corresponding prognosis information. FL achieved an AUC of 0.974 in renal cell carcinoma classification, outperforming all local models (with the highest at 0.943), and significantly predicted high-risk and low-risk patients. FL was shown to be effective for both classifying renal cancer and predicting survival in this study.

#### 3.8.5 Nasopharyngeal cancer

Nasopharyngeal carcinoma (NPC) is an epithelial malignancy originating from the nasopharyngeal mucosa, with approximately 130,000 new cases globally in 2020 (24). Clinically, contrast-enhanced T1-weighted MRI is highly effective in distinguishing NPC tumors. However, the gadolinium-based contrast agents used in imaging pose significant health risks. To address this, Li et al. (168) developed a FL framework named FL-TrustVCE for synthesizing virtual contrast-enhanced MRI (VCE-MRI) to replace scanned contrast-enhanced MRI. This framework combines the FLTrust method with a multimodality-guided synergistic neural network (169). They collected MRI data from 438 NPC patients across 18 institutions to validate the framework's effectiveness. The results demonstrated that their method achieved the best performance on both internal and external test sets compared to single-institution models, joint models, and two classic FL models, FedProx and FedAvg. Their approach successfully enhanced the generalization capability of the VCE-MRI model. The findings of this study highlight the positive impact of FL on the performance and generalization of cross-institutional VCE-MRI synthesis, marking a significant contribution to the field.

## 4 Discussion

### 4.1 Comparative analysis

We analyzed all 68 included studies with respect to three characteristics: the FL setting, external validation, and privacy strategy, as summarized in Table 10.

**Table 10:** . Summary of federated learning settings, external validation, and privacy strategies in the included studies.

Characteristic	Category	Number of papers (%)
FL setting	Simulated, artificial partition	34(50.0%)
	Simulated, site-based	32(47.1%)
	Real world distributed	6(8.8%)
External validation	Yes	12 (17.6%)
	No	56 (82.4%)
Privacy protection	Data localization only	36(52.9%)
	Differential privacy	7(10.3%)
	Homomorphic encryption	6(8.8%)
	Partial information sharing	16(23.5%)
	Blockchain	2(2.9%)
	Other	2(2.9%)

Categories are not mutually exclusive, as some studies may belong to multiple categories. Percentages are calculated based on the total number of included studies.

Most FL studies in cancer imaging were still conducted in simulated federated environments. Specifically, 50.0% of the studies artificially partitioned datasets into multiple virtual clients, with some further introducing heterogeneous data distributions through controlled partitioning to evaluate the robustness of their proposed methods. However, such artificially constructed heterogeneity may not fully reflect the complexity of real-world institutional differences. In comparison, 47.1% of the studies adopted site-based simulation, which better preserves institution-specific data distributions than artificial partitioning. Nevertheless, site-based simulation still cannot fully reproduce the challenges encountered during actual deployment, such as heterogeneous computing infrastructure, communication constraints, inter-institutional coordination, and differences in data standards. Only six studies (8.8%) conducted federated training in real-world distributed environments. These findings indicate that a substantial gap remains between methodological development and the real-world clinical deployment of FL in cancer imaging.

External validation is important for assessing model generalizability, particularly in medical imaging, where differences in patient populations, imaging scanners and acquisition protocols, and annotation practices can substantially affect model performance at unseen institutions. Among the 68 included studies, only 12 (17.6%) evaluated their models using an independent external institution or dataset, whereas the remaining 56 studies (82.4%) relied primarily on internal evaluation. This suggests that, although high performance has been reported in many studies, evidence supporting cross-institutional generalizability remains limited.

The main privacy strategies identified in FL studies of cancer imaging included data localization only, partial information sharing, differential privacy, homomorphic encryption, and blockchain-based approaches. Because one of the fundamental privacy advantages of FL is that raw data remain at local institutions, 52.9% of the included studies did not incorporate additional privacy-enhancing mechanisms beyond data localization. Partial information sharing was the second most frequently adopted strategy, accounting for 23.5% of the studies. By exchanging only selected model components, features, prompts, statistics, or other intermediate information, these approaches can reduce the amount of information exposed during collaboration while also lowering communication requirements. However, such strategies do not necessarily provide formal privacy guarantees comparable to dedicated privacy-enhancing techniques. Other measures included communication through a RabbitMQ/AMQP reverse proxy and peer anonymization. Differential privacy, homomorphic encryption, and blockchain-based approaches were adopted less frequently. Overall, these findings indicate that stronger and more rigorously evaluated privacy-enhancing mechanisms remain underexplored in FL research for cancer imaging.

### 4.2 Challenges of FL applying in cancer imaging

In the context of applying FL to cancer imaging, several significant challenges remain to be addressed. Among these, data security, data heterogeneity, and communication overhead are particularly prominent and require further attention.

#### 4.2.1 Data security

Even though FL avoids sharing raw local data, it only requires the local model parameters to be shared; some studies have indicated that certain attackers can infer partial participants’ original training data from the shared model parameters, posing privacy threats to the local data (170–172). To mitigate these potential data security issues in FL, several solutions have been proposed, including differential privacy and homomorphic encryption. Differential privacy, for instance, aims to enhance the protection of client data by introducing noise to the parameters uploaded by each client. However, one limitation of this method is that enhancing privacy often comes at the expense of model accuracy. The addition of noise may lead to a decrease in model performance (51, 173). Another solution is homomorphic encryption, in which model updates are encrypted before transmission and aggregated on the server in encrypted form. Although this reduces the risk of plaintext exposure, it introduces additional computational and communication overhead due to cryptographic operations, ciphertext expansion, and the greater complexity of computation in the encrypted domain (174).

#### 4.2.2 Data heterogeneity

Data heterogeneity in medical imaging, including feature distribution imbalances and quantity imbalances, presents significant challenges for FL (175). Variations in image quality, acquisition equipment, and scanning parameters among medical institutions can result in the distribution imbalances observed in medical image data. This results in data from different institutions being non-IID within the FL system. The amount of data held by different medical institutions can be unbalanced, further exacerbating these challenges (176). As a result, the presence of heterogeneous data presents a major problem to the effective implementation of FL in medical imaging. Methods such as data augmentation and regularization have been extensively explored to address the problem of data heterogeneity (177, 178). Data augmentation (179, 180) is a technique used to artificially enlarge a dataset by producing new samples derived from the original data. This technique can help balance imbalanced datasets, potentially improving the performance of FL models. However, certain data augmentation techniques in FL might require preliminary data sharing, which could increase privacy risks. On the other hand, incorporating regularization terms into FL algorithms can decrease the disparity between the global and local models. One such approach, FedProx (181), introduces proximal terms into the loss function to mitigate the effects of data heterogeneity. While effective, this method can also increase the complexity of model training, posing additional challenges in the implementation and optimization of FL systems.

#### 4.2.3 Communication overhead

In FL, local devices need to communicate with a central server to transmit model parameters and updates. When training FL models on a large scale with multiple institutions and a substantial amount of cancer image data, communication overhead can become a problem. The involvement of numerous clients in training increases communication costs. Additionally, limited or unstable network bandwidth can cause delays in model parameter transmission, thereby affecting or interrupting FL training (182). Overcoming communication bottlenecks is a complex and ongoing task. In response to the communication challenges within FL environments, various solutions aimed at enhancing communication efficiency have been extensively studied, such as machine learning compression technologies (182). Compression technology in FL includes the compression of both the model and the training process, which can reduce the overall communication costs (183). However, this may come at the expense of losing some training accuracy. For instance, Haddadpour (184) and his team employ regularly compressed (quantized or sparse) communication to optimize FL processes. For environments with homogeneous data distributions, they developed the FedCOM algorithm, which not only enhances the existing convergence bounds but also offers more rigorous and stringent convergence estimates for both strongly convex and non-convex objective functions. For heterogeneous local distributions, they introduced the FedCOMGATE algorithm, which effectively handles data heterogeneity between different devices by introducing a local gradient tracking mechanism.

Despite the promising results were shown, substantial efforts are warranted to further improve the performance of FL in these three aspects.

### 4.3 Future research directions of FL in cancer imaging

#### 4.3.1 Designing effective incentive mechanisms

Medical data is highly valuable and sensitive, institutions that own such data may hesitate to engage in FL without clear incentives. This is often due to concerns about intellectual property rights, data security, and the resource demands associated with participating in training (185). Therefore, establishing a reasonable incentive mechanism is crucial to encourage institutions to participate actively (186). Effective incentive mechanisms not only provide tangible benefits to participating institutions, attracting more participants but also include negative incentives to penalize malicious behavior, such as actions that could degrade the performance of the FL model. Such measures are vital for identifying and excluding malicious institutions and enhancing the FL model performance (187).

#### 4.3.2 Personalized FL for cancer imaging

A key challenge in medical imaging is data heterogeneity, primarily because of the diverse imaging equipment and protocols used by various medical institutions. This can affect the model's generalization trained through traditional FL methods. In response, PFL has emerged as a promising solution, allowing each participating client to develop and refine its own local model tailored specifically to its dataset. PFL is helpful for improving model adaptability to local conditions and effectively reduces issues caused by data heterogeneity (188). Despite recent initiatives, such as (35, 60, 132, 137), exploring the potential of PFL in cancer imaging, the field is still at an early stage and requires more in-depth study.

#### 4.3.3 Open multi-institutional medical image datasets

The development and validation of FL algorithms in the medical imaging domain critically depend on access to multi-institutional datasets. High-quality public datasets are particularly valuable as they enable researchers worldwide to collaboratively improve algorithmic performance. However, such datasets are scarce and often restricted to specific anatomical regions, such as BraTS and IXI, possibly due to the complexities of multi-institutional data sharing agreements across institutions and patient consent. To promote the application and innovation of FL in cancer imaging, it is imperative to establish a larger number of public multi-institutional medical image datasets, which reflect the broad spectrum of clinical settings, imaging modalities, and patient populations.

#### 4.3.4 Expand FL applications in cancer imaging

In the articles reviewed in this study, we found that majority of research focuses on tumor classification, segmentation, and detection, among other area. This research tendency may be due to the availability of well-established benchmark datasets and related studies in these areas, which facilitate the validation of FL frameworks. However, to maximize the impact of FL in cancer imaging, it is necessary to expand research into new domains, such as conversion between image modalities (189, 190), image reconstruction (191, 192), treatment planning (193, 194), radiotherapy dose prediction (195, 196), predicting treatment outcomes and survival rates (197, 198), and radiogenomics for integrative analytics (199). Research in the above directions not only helps overcome the data limitations faced by traditional techniques, but also promote the development of FL from auxiliary diagnosis to full-process intelligent decision-making.

#### 4.3.5 Clinical translation and real-world deployment

As FL methodologies continue to mature, clinical deployment is becoming increasingly feasible. However, translating FL from methodological research into routine clinical practice still requires several practical challenges to be addressed. One major issue is the coordination of participating institutions with different dataset sizes, computational resources, and network conditions. Under synchronous aggregation, slower clients can delay the progress of each training round. In a distributed melanoma pathology study, Schoenpflug et al. (41) reported that experiments required several days because of the large volume of image data and heterogeneity in client resources. Considerable effort was also required for network configuration, task orchestration, and experiment logging. These observations indicate that future FL systems should incorporate more efficient and flexible mechanisms for coordinating training across institutions with heterogeneous resources.

Technical coordination also needs to be supported by appropriate governance arrangements among participating institutions. Responsibilities for network access, initiation of training, approval of model updates, and responses to security or performance issues should be clearly defined, while training logs should be maintained to support the management and traceability of experimental cycles. Clinical deployment must also comply with ethical and legal requirements related to patient privacy. As participating institutions may operate under different regulatory frameworks, clear mechanisms for participation agreements, access control, and traceability are required. The European Cancer Imaging Initiative (EUCAIM) (200) provides an example of such an infrastructure by incorporating participation agreements, access review, and traceable records.

Another important gap is the limited availability of prospective validation. Most existing FL studies are retrospective, making it difficult to determine whether performance observed during experimental evaluation can be maintained after deployment. In real clinical settings, local data distributions, participating institutions, and model versions may change over time, and the effect of these changes on federated models remains insufficiently evaluated. Future research should therefore place greater emphasis on prospective studies that assess FL under evolving clinical conditions.

Overall, advancing the clinical translation of FL will require greater attention to cross-institutional coordination, governance, regulatory compliance, and prospective validation. Addressing these issues will be essential for moving FL beyond retrospective and experimental studies toward reliable use in real-world clinical environments.

#### 4.3.6 Emerging AI models and parameter-efficient federated learning

Recent advances in learning paradigms and foundation models have created new opportunities for the development of FL in cancer imaging. Conventional FL methods, such as FedAvg and FedProx, generally assume that participating institutions use models with identical architectures, allowing model parameters to be directly aggregated. However, real-world medical environments often differ substantially in computational resources, data distributions, and locally adopted model architectures, creating a need for FL frameworks that can accommodate heterogeneous local models (178). This has driven growing interest in heterogeneous federated learning (201). Federated knowledge distillation offers one potential solution by enabling collaborative learning through the exchange of model knowledge rather than direct parameter aggregation (202). The transferred knowledge may take different forms, including response-based, feature-based, and relation-based representations. Dataset distillation provides another approach by allowing institutions to share compact synthetic data that retain informative characteristics of their local datasets, thereby supporting collaboration without requiring identical local model architectures (77). Together, these strategies increase the flexibility of FL and facilitate collaborative learning across institutions with heterogeneous models.

Foundation models represent another important direction for FL (203). By learning generalizable representations from large-scale datasets and adapting them to downstream tasks with limited local data, foundation models are particularly relevant to medical applications, where annotated data are often scarce. Recent studies have begun to integrate vision foundation models into FL through parameter-efficient prompt learning. For example, Fed-GPL collaboratively trains a prompt generator to produce patient-specific prompts for adapting foundation models to different medical imaging tasks, while updating only a small subset of the foundation model parameters (204). However, the large size of foundation models introduces substantial computational and communication costs. This has further motivated the use of parameter-efficient fine-tuning strategies, including prompt-based and adapter-based learning, in which only a small subset of trainable parameters is updated and communicated while most pretrained parameters remain frozen (203). This can substantially reduce communication overhead. Future research should further explore how emerging AI models and FL paradigms can be jointly developed to balance model performance, communication efficiency, privacy protection, and adaptability in heterogeneous cross-institutional environments.

#### 4.3.7 Multimodal federated learning for cancer imaging

Cancer diagnosis and treatment often rely on complementary information from multiple sources, including CT, MRI, histopathological images, clinical data, and genomic information. In the broader field of medical AI, multimodal learning has been widely explored by integrating multiple imaging modalities with clinical and other non-imaging data, and such approaches have been shown to improve model performance (205). In contrast, most existing FL studies in medical imaging remain focused on a single modality, with relatively few studies investigating multimodal federated settings (206). Multimodal FL, however, introduces additional challenges. Participating institutions may have access to different combinations of modalities, while some modalities may be missing, unpaired, or unevenly distributed across clients. In addition, substantial differences in feature representations across imaging, pathological, clinical, and genomic data increase the complexity of cross-modal feature fusion and federated aggregation (207). Future research should therefore focus on developing flexible multimodal FL frameworks that can effectively learn from heterogeneous and incomplete modality combinations across institutions. Such frameworks could facilitate more comprehensive use of distributed clinical information and support the development of federated cancer imaging models with improved robustness and generalizability.

## 5 Conclusion

This article presented a comprehensive review of the application of FL in cancer imaging. First, we analyze the existing review articles on FL to distinguish our contribution from previous surveys. Then, we outline the concept and basic paradigm of FL. We review FL research in medical imaging for 11 common types of cancer, summarizing the main contributions and methods. We then highlight the challenges of FL in cancer imaging including data security, data heterogeneity, and communication overhead, along with potential solutions to address these challenges. Finally, we provide perspectives on possible future research directions in cancer imaging, which are vital for advancing the field.
