# Supplementary material for: Federated learning in cancer imaging: a comprehensive review and future outlook
Source: Front Radiol. 2026 Oct 28;6:1976540. doi: 10.3389/fradi.2026.1976540 (PMC13752500; doi:10.3389/fradi.2026.1976540)
Supplement: Supplementary file 1 [file Table1.pdf]

Supplementary Tables

Detailed study-level characteristics of the federated learning studies included in this review are provided in Supplementary Tables S1–S7. The tables report information on the clinical task, imaging modality, federated learning method, dataset, number of clients, experimental setting, model architecture, main results, and privacy strategy.

Supplementary Table S1. Detailed characteristics of federated learning applications for brain cancer

Task	Ref	Modality	FL method	Dataset	Clients	Setting	Model	Main result	Privacy strategy
**Segmentation**	[28]	MRI	FedAvg	BraTS 2018	10/50/100	Simulated, artificial partition	U-Net	Dice 0.89, sens. 0.92, spec. 0.96	Differential privacy, encryption, secure aggregation
	[20]	MRI	Synchronous weighted FedAvg	BraTS 2018	13	Simulated, site-based	3D segmentation network	FL achieved performance comparable to centralized training.	Data locality, selective parameter sharing, gradient clipping, Sparse Vector
	[32]	MRI	FedAvg	BraTS 2017; MDACC and WashU	10	Simulated, site-based	U-Net	BraTS: Dice 0.86; WashU: Dice 0.77; MDACC: Dice 0.82	Data locality only
	[33]	MRI	SplitAVG; SplitAVG-v2	BraTS 2017	10	Simulated, site-based	U-Net	Dice: 84.6%	Data locality; intermediate-feature communication; SplitAVG-v2 keeps labels local
	[34]	MRI	FL	BraTS 2020	17	Simulated, site-based/artificial partition	3D U-Net	Visible-test Dice 0.67; hidden-test Dice 0.62 / 0.61	Data locality only
	[35]	MRI	CFFT	FeTS 2022 / BraTS 2021	23	Simulated, site-based	3D U-Net	Dice: 0.89, HD95: 5.8749	Data locality, radiomic-feature sharing
	[38]	MRI	Vanilla and decentralized FL tools based on MQTT	BraTS 2018; BraTS 2020; private Athens MRI dataset	5	Real world distributed	U-Net	Dice >0.85	Data locality, TLS-encrypted/authenticated communication
**Classification**	[43]	MRI	FedAvg	UK Data Service dataset; extended Kaggle dataset	50	Simulated, artificial partition	CNN ensemble (DenseNet121, VGG19, InceptionV3)	UK dataset: Acc. 91.05%, AUC 0.91; extended dataset: Acc. 93.22%	Data locality only

	[40]	MRI	Average-weight FL	BT-small-2c; BT-large-3c	2	Simulated, artificial partition	CNN	Acc. 82% / 96%, F1 87% / 97%	Data locality only
	[19]	MRI	FedAvg; QFedAvg; Ft-FedAvg; Dp-FedAvg	Brain Tumor MRI Dataset	3	Simulated, artificial partition	CNN	10 rounds: Acc. 85.55%; 20 rounds: Acc. 84.60%; 30 rounds: Acc. 85.80%	Data locality; differential privacy in the Dp-FedAvg experiment
	[39]	MRI	Collaborative transfer-learning FL	Open-source four-class brain MRI dataset	4	Simulated, artificial partition	DenseNet201	IID: Acc. 96.39%; Non-IID: Acc. 94.44%	Data locality only
**Reconstruction**	[46]	MRI	FedGIMP	IXI; fastMRI; BraTS; Bilkent in-house dataset	3	Simulated, site-based	Unconditional adversarial model	Outperformed the second-best FL method by 3.7 dB in PSNR and 3.0% in SSIM on average.	Data locality; local discriminators remain site-specific;
	[47]	MRI	FL-MRCM	fastMRI; HPKS; IXI; BraTS	4	Simulated, site-based	U-Net	T1 SSIM 0.94, PSNR 34.14 dB; T2 SSIM 0.94, PSNR 33.13 dB	Data locality,latent-feature sharing;

Supplementary Table S2. Detailed characteristics of federated learning applications for lung cancer

Task	Ref	Modality	FL method	Dataset	Clients	Setting	Model	Main result	Privacy strategy
Classification	[50]	CT	FedAvg	Lung cancer datasets from five clients	5	Simulated, site-based	CNN	Acc. > 95%	Data locality only
	[51]	Histopathology	FedAvg; DP-FL	TCGA LUAD/LUSC WSI	4/8/16/32	Simulated, artificial partition / site-based	MEM model	ε=2.90, δ=0.0001: Acc. 82.3%; external hospital: Acc.70.7%	Differential privacy（DP-SGD with Gaussian noise）
	[53]	Histopathology	CIT2FR-FL-NAS	LC25000	3	Simulated, artificial partition	CNN	Highest-complexity: Acc. 99.96%; minimum-complexity: 92.98%	Data locality only
	[55]	Histopathology	Hyper-MIL	TCGA-NSCLC; PANDA	8	Simulated, artificial partition	Attention-based MIL; server hyper-network	TCGA-NSCLC: AUC 0.92, error 0.21, bAcc. 0.86, F1 0.88	Data locality, noise-added parameter sharing
	[56]	Histopathology	DCIaaS	LC25000	5	Simulated, artificial partition	CNN	Acc. 96.52%	Data locality, blockchain-based parameter sharing
	[57]	CT	FedAvg	Chest CT-Scan Images Dataset（Kaggle）	2/3/4/5	Simulated, artificial partition	Lung-AttNet	2/3 clients: Acc. 92%; 4 clients: Acc. 82%; 5 clients: Acc. 67%	Data locality only
	[60]	Histopathology	PFL	LC25000	10	Simulated, artificial partition	Convolutional autoencoder	Acc. 100.0%, Sens. 1.00, Spec. 1.00; AUC 1.00	Data locality; encoder weights/model updates shared
	[61]	CT	FL-CNN	Lung CT datasets from six medical institutions	6	Simulated, site-based	CNN	Per-client: Acc. 89.02%–92.81%; macro-average: 88.40%–92.67%	Data locality only
Detection	[63]	CT	FedLRes	IQ-OTH/NCCD	4	Simulated, artificial partition	ResNet50	Acc. 99.40%, Prec. 98.92%, Spec. 99.36%	Data locality only
	[66]	CT	FBCLC-Rad	Cancer Imaging Archive; Kaggle Data Science Bowl 2017; LUNA16; local dataset	8	Simulated, artificial partition	CapsNet	Acc. 99.69%, F1 99.1%, Spec. 99.4%	Data locality, blockchain-based parameter sharing/authentication

Supplementary Table S3. Detailed characteristics of federated learning applications for breast cancer

Task	Ref	Modality	FL method	Dataset	Clients	Setting	Model	Main result	Privacy strategy
Classification	[73]	Histopathology	FedAvg	BreakHis	11	Simulated, artificial partition	ResNet152; DenseNet201; MobileNet-v2-100; EfficientNet-B7	Image-level Acc. 91.06%; patient-level Acc. 91.87%; F1 84.97%	Homomorphic encryption（CKKS）for model parameters, AES for service requests/results
	[74]	Histopathology	FedAvg	BHI, BreakHis	3	Simulated, artificial partition	ResNet, GaborNet	BHI: Acc. 86.57%, F1 86.49%; BreakHis: Acc. 86.32%, F1 87.4%	Data locality only
	[75]	Imaging tabular cytological features	FedAvg	Breast Cancer Wisconsin Diagnostic	10	Simulated, artificial partition	Fully connected feed-forward neural network	FL+DP: Acc: 96.1%;FL without DP: 97.7%	Differential privacy
	[76]	Histopathology	SiloBN	CAMELYON16; CAMELYON17	7	Simulated, site-based	DCNN	CAMELYON16: AUC 0.94; CAMELYON17: AUC 0.88	Data locality, Keep BN statistics local
	[77]	Histopathology	FedWSIDD	CAMELYON16; CAMELYON17	7	Simulated, site-based	ResNet50	CAMELYON16: Acc. 90.1%; CAMELYON17: Acc. 81.2%	only distilled synthetic slides are exchanged.
	[78]	Histopathology	HistoFS	TCGA-RCC; HER2; CAMELYON17	3	Simulated, site-based	SSL-ViT encoder with an MIL classifier	RCC: Auc 98.11%; HER2: Auc 86.07%	Data locality only; pseudo-bag styles and the MIL weight matrix WW are shared.
	[79]	Histopathology	FedDFP	CAMELYON16; CAMELYON17; TCGA-IDH	15	Simulated, site-based	Heterogeneous feature extractors and MIL classifiers	CAMELYON16: Acc. 90.1%;CAMELYON17: Acc. 91.5%; TCGA-IDH: Acc. 94.3%	Data locality only; only compact deep feature prompts are shared.
	[80]	Histopathology	FedHD	CAMELYON16; CAMELYON17; TCGA-IDH	15	Simulated, site-based	ResNet-50, UNI, PhikonV2, and GPFM feature extractors; CLAM, TransMIL, and ACMIL classifiers	CAMELYON16: Acc. 91.2%;CAMELYON17: Acc. 82.7%; TCGA-IDH: Acc. 84.8%	Only distilled feature tensors and labels are shared

	[81]	Mammography	HDRO-FL	CBIS-DDSM	-	Simulated, artificial partition	DenseNet, E-RNN	Acc. 95.7%; Sens. 95.7%; Spec. 95.73%; F1 95.8%	Data locality only
	[82]	Mammography	FedAvg	Private mammography data from seven international institutions	7	Real-world distributed	DenseNet121	Mean within-site Cohen's κ: 0.68	Data locality, SSL/TLS-encrypted communication
**Detection**	[93]	Histopathology	FedAvg	CAMELYON16	4/3	Simulated, artificial partition	CNN	4 client: AUC 98.2%; 3 client: AUC 98.03%	Data locality only
**Segmentation**	[94]	ultrasound	FedMix	BUS; BUSIS; UDIAT	3	Simulated, site-based	U-Net	Semi-supervised: Dice 80.6%; mixed supervision: Dice 83.2%	Data locality only
	[98]	mpMRI	Cross-domain FL with FedAvg	Breast mpMRI	2	Simulated, artificial partition	U-Net	Dice: 0.65	Data locality only
	[100]	Histopathology	TheODen	BCSS; SemiCOL	3	Real world distributed	U-Net	BCSS: Dice 0.76; SemiCOL: Dice 0.75	Data locality; communication through a RabbitMQ/AMQP reverse proxy

Supplementary Table S4. Detailed characteristics of federated learning applications for skin cancer

Task	Ref	Modality	FL method	Dataset	Clients	Setting	Model	Main result	Privacy strategy
Classification	[102]	Dermoscopy	Async-FL-CNN	ISIC 2019	5	Simulated, artificial partition	CNN	Acc. 93.4%, F1 94.8%, Sens. 94.1%, Spec. 96.3%	Data locality only
	[103]	Dermoscopy	Adaptive federated machine learning	ISIC 2019	-	Simulated, artificial partition	CNN	Acc. 95.6%, Prec. 0.95, recall 0.95	Data locality only
	[104]	Dermoscopy	FedML	HAM10000	1/2/3/4/5	Simulated, artificial partition	CNN	Acc. 90.1%	Data locality only
	[108]	Dermoscopy	FedAvg	SIIM, ISIC	5	Simulated, artificial partition	EfficientNet	Acc. 83.01%, F1 84.02%, AUC 0.94	Data locality only
	[109]	Dermoscopy	RePFL	HAM10000	20	Simulated, artificial partition	ResNet-50	Acc. 87.5%/86.86% under two extreme non-IID settings	Data locality only
	[110]	Dermoscopy	Async-FL	ISIC 2019	4	Simulated, artificial partition	CNN, SVM	Acc. >92%	Data locality only
	[111]	Dermoscopy	Privacy-preserving FL	HAM10000; ISIC 2019	5	Simulated, artificial partition	DNN	HAM10000: Acc. 88.27%; ISIC 2019: Acc 83.07%	Fully homomorphic encryption; layer-wise packed FHE
	[112]	Dermoscopy	FedPerl	ISIC19, HAM, Derm7pt, PAD-UFES	10	Simulated, artificial partition	EfficientNet	F1 0.75, precision 0.76, recall 0.75	Data locality, peer anonymization
	[115]	Dermoscopy	Federated contrastive learning	ISIC 2019; AtlasDerm; Dermnet; DarkDerm	10	Simulated, artificial partition	ResNet18	Recall 59.23%, Prec. 50.21%	Data locality, server-de-identified feature sharing
	[119]	Histopathology	FedAvg	Slides of skin lesions from 6 German university hospitals	5	Simulated, site-based	ResNet18	Federated test set: AUC 0.86; the sixth-hospital external test set: AUC 0.91	Data locality only

Supplementary Table S5. Detailed characteristics of federated learning applications for prostate cancer

Task	Ref	Modality	FL method	Dataset	Clients	Setting	Model	Main result	Privacy strategy
Segmentation	[124]	MRI	FedAvg	Multi-center private prostate MRI dataset	8	Simulated, site-based	Two-Stage Cascaded U-Net	Dice 0.84, Jaccard: 0.72	Data locality only
	[120]	MRI	FedAvg	Private MRI from UCLA, SUNY Upstate, and NCI; PROSTATEx	3	Real world distributed	3D AH-Net	private test sets: Dice 0.90; PROSTATEx: Dice 0.89	Data locality, SSL/TLS-encrypted communication
	[125]	MRI	FedAvg	MSD-Prostate; PROMISE12; NCI-ISBI13; ProstateX	4	Simulated, site-based	AutoML	Dice 0.89	Data locality only
	[129]	MRI	HarmoFL	NCI-ISBI 2013, I2CVB, PROMISE12	6	Simulated, site-based	U-Net	Dice 94.28%	Data locality only
	[132]	MRI	Personalized retrogress-resilient FL	NCI-ISBI 2013; PROMISE12	5	Simulated, site-based	Deeplab-v2, ResNet-18	Dice 78.28%, IoU 71.92%	Data locality; high-frequency client-specific parameter components excluded from aggregation
Classification	[137]	MRI	CusFL	PROSTATEx; LocalPCa (private)	2 / 4 / 8 / 16 / 32	Simulated, site-based / artificial partition	Custom network	2-client setting: private/public Avg. Acc. 87.5% / 80.5%; private/public Avg. AUC 95.6% / 88.7%	Data locality; personalized model parameters remain local
	[138]	MRI	VAFL	LocalPCa; PROSTATEx	8/16	Simulated, site-based / artificial partition	Custom network	8-client VAFL: overall Acc. 0.98; AUC 0.998	Differentially private gradient-noise training in PPWGAN-GP, filtered synthetic-image sharing
	[139]	MRI	FedAvg	TCIA PROSTATE-MRI	100	Simulated, artificial partition	SBEROA-optimized ShPyFHNet	Acc. 96.75%, MAP 96.4%	Data locality only

Supplementary Table S6. Detailed characteristics of federated learning applications for pancreatic cancer

Task	Ref	Modality	FL method	Dataset	Clients	Setting	Model	Main result	Privacy strategy
**Segmentation**	[148]	CT	DTP, DWA	TCIA; MSD; Synapse	3	Simulated, site-based	C2FNAS	DTP: Dice 46.1%; DWA: Dice 61.4%	Data locality, percentile sharing protocol: only the 25% largest absolute model updates are shared
	[144]	CT	Auto-FedAvg	MSD, TCIA, BTCV	3	Simulated, site-based	3D U-Net	Dice 74.21%	Data locality only; differential privacy was discussed only as future additional protection
	[143]	CT	Horizontal FL	Abdominal CT images from two multinational institutions	2	Real world distributed	C2FNAS	Dice: C1 pancreas 82.3%; C2 pancreas 65.4%; C2 tumor 46.4%; pancreas average 73.1%	Data locality, token-based authentication, SSL-secured gRPC communication
	[149]	CT	FedRNN	MSD, Pancreas-CT, BTCV	3	Simulated, site-based	Elman RNN	Dice 78.7%	Data locality, percentile sharing protocol: only the 25% largest absolute model updates are transmitted

Supplementary Table S7. Detailed characteristics of federated learning applications for other cancer

Task	Ref	Modality	FL method	Dataset	Clients	Setting	Model	Main result	Privacy strategy
**Colorectal classification**	[151]	Histopathology	FedAvg	LC25000	2	Simulated, artificial partition	VGG 16 network	Lung: Acc. 99.99%; colon: Acc. 99.72%	Data locality only
	[152]	Histopathology	FedAvg	NCT-CRC-HE-100K	10	Simulated, artificial partition	CNN	Acc. 96.04%	Data locality only
	[154]	Histopathology	FedDropoutAvg	TCGA CRC-DX dataset	11	Simulated, site-based	ResNet18	Independent sites: F1 0.91, AUC 0.95; local test sites: F1 0.85, AUC 0.97	Data locality only; client/parameter dropout
**Liver segmentation**	[155]	CT, MRI	FedNorm / FedNorm+	LiTS17, 3D-IRCADb-01, Multi-Atlas, SLIVER07, and CHAOS19.	6	Simulated, site-based	U-Net	CT: Dice 0.96; MRI: Dice 0.94	Data locality only
	[159]	CT	Fed-MENU	LiTS, KiTS, pancreas, spleen, AMOS, BTCV	5	Simulated, site-based	MENU-Net	Dice 91.14%	Data locality only
**Thyroid classification**	[165]	Ultrasound	FL	Thyroid ultrasound images from six institutions	6	Real world distributed	VGG19, ResNet50, ResNext50, SE-ResNet50, SE-ResNext50	Internal: Acc. 73.9%–79.5%; external: Acc. 56%–76%	Data locality only
**Kidney classification**	[166]	Histopathology	Prop-FFL	Kidney Dataset from TCGA	4	Simulated, site-based	DenseNet121	Acc. 86.7%	Data locality only
	[167]	Histopathology	Weakly supervised FedAvg with Gaussian parameter noise	Kidney Dataset from TCGA	7	Simulated, site-based	Multiple instance learning-based framework	BRCA: AUC 0.833–0.862; Rcc: AUC 0.974–0.976	Data locality, Gaussian noise added to model parameters
**Nasopharyngeal synthesis**	[168]	MRI	FL-TrustVCE	MRI dataset from 18 institutions	3	Simulated, site-based	MMgSN-Net	Internal: MAE 23.45, PSNR 33.23; external MAE 30.86, PSNR 31.87	Data locality for local clients, FLTrust trust

									scoring, update normalization and clipping
